# Applications of nanotechnologies for miRNA-based cancer therapeutics: current advances and future perspectives

**DOI:** 10.3389/fbioe.2023.1208547

**Published:** 2023-07-27

**Authors:** Luis Alberto Bravo-Vázquez, Andrea Méndez-García, Alma L. Rodríguez, Padmavati Sahare, Surajit Pathak, Antara Banerjee, Asim K. Duttaroy, Sujay Paul

**Affiliations:** ^1^ Tecnologico de Monterrey, School of Engineering and Sciences, Querétaro, México; ^2^ Instituto de Neurobiología, Universidad Nacional Autónoma de México, Querétaro, México; ^3^ Department of Medical Biotechnology, Faculty of Allied Health Sciences, Chettinad Academy of Research and Education (CARE), Chettinad Hospital and Research Institute (CHRI), Chennai, India; ^4^ Department of Nutrition, Institute of Basic Medical Sciences, Faculty of Medicine, University of Oslo, Oslo, Norway

**Keywords:** MicroRNAs, cancer, nanoparticles, therapeutics, gene regulation, targeted delivery

## Abstract

MicroRNAs (miRNAs) are short (18–25 nt), non-coding, widely conserved RNA molecules responsible for regulating gene expression via sequence-specific post-transcriptional mechanisms. Since the human miRNA transcriptome regulates the expression of a number of tumor suppressors and oncogenes, its dysregulation is associated with the clinical onset of different types of cancer. Despite the fact that numerous therapeutic approaches have been designed in recent years to treat cancer, the complexity of the disease manifested by each patient has prevented the development of a highly effective disease management strategy. However, over the past decade, artificial miRNAs (i.e., anti-miRNAs and miRNA mimics) have shown promising results against various cancer types; nevertheless, their targeted delivery could be challenging. Notably, numerous reports have shown that nanotechnology-based delivery of miRNAs can greatly contribute to hindering cancer initiation and development processes, representing an innovative disease-modifying strategy against cancer. Hence, in this review, we evaluate recently developed nanotechnology-based miRNA drug delivery systems for cancer therapeutics and discuss the potential challenges and future directions, such as the promising use of plant-made nanoparticles, phytochemical-mediated modulation of miRNAs, and nanozymes.

## 1 Introduction

Cancer is a multifactorial disease distinguished by altered cell pathways and complex molecular mechanisms leading to unrestrained growth and proliferation of cells (Geraldelli et al., 2020; Javed Iqbal et al., 2021). Despite the latest advances in cancer diagnosis and therapies, the global burden of cancer is still significant and is forecasted to increase in the following years (Pilleron et al., 2021; De Silva and Alcorn, 2022). Further, at least 1,958,310 novel cancer cases and 609,820 cancer fatalities are estimated to occur in the United States in 2023 (Siegel et al., 2023); while 34 million new cancer cases are predicted to be diagnosed globally in 2070 (Soerjomataram and Bray, 2021).

As a result, developing a successful strategy to prevent and/or treat cancer has become one of the main goals of our era. Among the most outstanding approaches that have lately been developed with this purpose are next-generation probiotics (Kaźmierczak-Siedlecka et al., 2022), immune cell-derived extracellular vesicles (Yang et al., 2021b), mitochondria targeting strategies (Ghosh et al., 2020), tumor necrosis factor (TNF)-related apoptosis-inducing ligand (TRAIL)-targeting strategies (Kretz et al., 2019), anti-angiogenic therapies (Teleanu et al., 2020), anti-cancer vaccines (Rahimian et al., 2021; Saxena et al., 2021), and drugs targeting cancer metabolism (Kim, 2019). In particular, the current progress in nanomedicine has provided promising options for cancer therapy by exploiting the physicochemical features of nanocarrier-based drug delivery systems, which could help improve the efficacy of cancer chemotherapy and mitigate its associated unwanted effects (Giustarini et al., 2021; Wei et al., 2021; Wu et al., 2022). It is worth mentioning that, along with nanomedical devices, miRNAs have emerged as potential therapeutic agents against cancer (Mollaei et al., 2019; Tao et al., 2020; Holjencin and Jakymiw, 2022).

**FIGURE 1:**
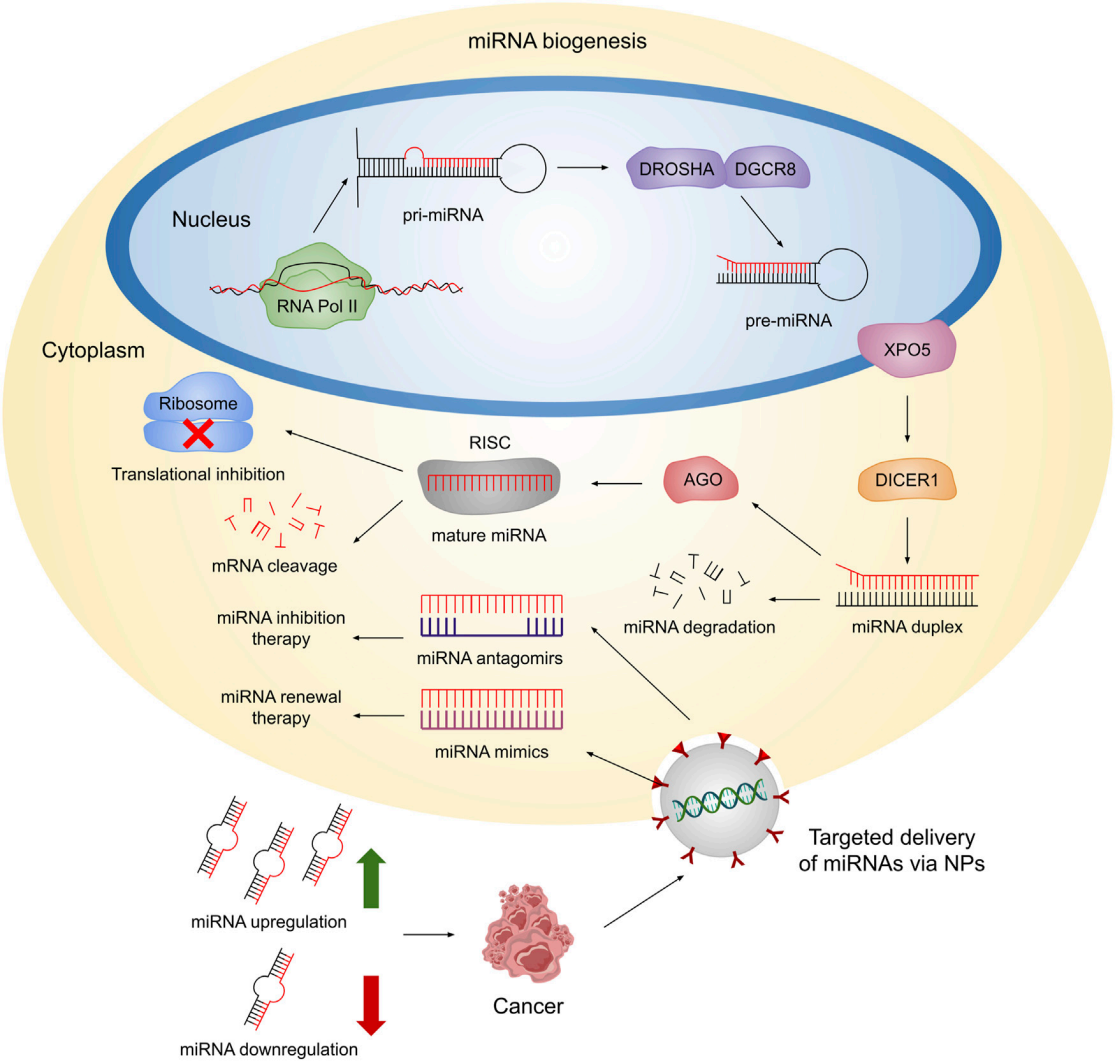
Schematic representation of miRNA biogenesis. In the nucleus, RNA Pol II transcribes miRNAs, which are then folded into a secondary hairpin shape and stem-loop structure called pri-miRNA. Afterward, such structure is cleaved by the RNases II DROSHA and DGCR8, to generate pre-miRNAs. Subsequently, XPO5 transports pre-miRNAs to the cytoplasm, where DICER1 processes them to form a miRNA duplex, composed of a guide and passenger strand. The latter is degraded while the guide strand binds to AGO in order to form the RISC. Finally, mature miRNAs are able to regulate gene expression at the post-transcriptional level by mRNA cleavage or translation inhibition. Dysregulation of endogenous miRNA expression can contribute to the progression of cancer. Hence, therapeutic approaches employing NPs provide an opportunity for targeted delivery of antagomirs or mimics to the cytoplasm. This targeted delivery allows for the inhibition of endogenous miRNAs or restoration of miRNA function, respectively, aiming to restore miRNA levels and promote a healthy state.

MicroRNAs (miRNAs) are a class of tiny (18–25 nucleotides), endogenous, extensively conserved, non-coding transcripts that regulate gene expression at the post-transcriptional level (Damase et al., 2021; Sidorova et al., 2023). MiRNA biogenesis begins in the nucleus, where the RNA polymerase II produces 500–3,000 nucleotides-long primary miRNAs (pri-miRNAs). Subsequently, pri-miRNAs acquire a secondary structure of hairpin-shape and stem-loop that is then cleaved by the RNase III family enzymes DROSHA and RNA binding protein DiGeorge syndrome critical region 8 (DGCR8) in order to generate precursor miRNAs (pre-miRNAs) (Ding et al., 2019; Ali Syeda et al., 2020). Exportin 5 (XPO5) then transports pre-miRNAs from the nucleus to the cytoplasm, where they are further processed by DICER1 to create a miRNA duplex (Ding et al., 2019; Ali Syeda et al., 2020). The miRNA duplex comprises a guide and passenger strand, which are then separated by RNA helicase. Afterward, the guide strand binds the Argonaute (AGO) protein to assemble the RNA-induced silencing complex (RISC), while the passenger strand is usually degraded (Salamati et al., 2020). Finally, miRNAs mediate post-transcriptional gene silencing by interacting with the 3′-untranslated region (UTR) of their mRNA targets, leading to either mRNA cleavage or translational inhibition (Pu et al., 2019). The biogenesis pathway of miRNAs is depicted in Figure 1.

Since miRNAs are involved in several biological processes, their dysregulation gives rise to various health disorders, including cancer (Paul et al., 2020a; Paul et al., 2020b; Paul et al., 2020c; Paul et al., 2021a; Paul et al., 2021b; Bravo-Vázquez et al., 2022; Paul et al., 2022; Ruiz-Manriquez et al., 2022; Bravo-Vázquez et al., 2023a; Bravo-Vázquez et al., 2023b). In fact, miR-9, miR-10b, miR-17, miR-21, miR-132, miR-155, miR-222, miR-375, and miR-519a have been identified as oncogenes (oncomiRs) (Fu et al., 2021). Consequently, artificial miRNAs have arisen as therapeutic devices aimed at reestablishing the physiological expression levels of these molecules in different diseases. For instance, miRNA mimics can be employed to restore miRNA levels in cases when diminished miRNA expression is the cause of the disorder (Dasgupta and Chatterjee, 2021; Bibby et al., 2022); while anti-miRNAs (antagomirs) are used to block the activity of disease-causing overexpressed miRNAs via sequestering miRNAs away from their mRNA targets (Dasgupta and Chatterjee, 2021; Bibby et al., 2022). Despite this, many challenges remain to be solved for the effective *in vivo* delivery of miRNA mimics and antagomirs. For example, both miRNA mimics and antagomirs do not easily penetrate into tumor tissues, and naked miRNA mimics and antagomirs are easily degraded in the bloodstream due to the presence of nucleases (Chen et al., 2015). Furthermore, potential neurotoxicity and immunotoxicity, adverse effects, and off-target effects are factors that must be taken into account in the design of miRNA-based cancer therapies (Chen et al., 2015).

Hence, nanoparticles (NPs) have been harnessed in multiple studies to efficiently deliver miRNA-centered drugs into cells and/or model organisms of different types of cancer alone or in conjunction with chemotherapeutic drugs to achieve a synergistic effect with better results in cancer management (He et al., 2020). Some examples of tumor suppressor miRNAs that have been employed in NP-based therapies are let-7a/b, miR-29b, miR-34a, miR-100, miR-122, miR-133b, miR-204-5p, and miR-634 (O’Neill and Dwyer, 2020). Therefore, in this brief review, we present an overview of the most recent advances in the application of NPs for the delivery of miRNA-based drugs against different types of cancer; as well, we discuss the future prospects of these next-generation therapies.

## 2 Advantages of nanotechnology-mediated miRNA-based drug delivery in cancer

Throughout the last decade, outstanding advances have been made in the development of nanotechnology-based strategies for cancer drug delivery, particularly for breast cancer (Raikwar et al., 2022), lung cancer (Li et al., 2023), and glioblastoma (Liu et al., 2023). Remarkably, the global market value of nanomedicine was estimated at 138.8 billion USD in 2016, and it is expected to be worth 350.8 billion USD by 2025 (Halwani, 2022). A number of favorable outcomes have been obtained experimentally from the NP-mediated delivery of non-coding RNAs (ncRNA), including miRNAs and small interfering RNAs (siRNAs), into cancer stem cells and metastatic tumors, leading to enhanced and efficient treatment options for cancer (Alzhrani et al., 2020). In addition, the approval of the distribution in the pharmaceutical market of nanotechnology platforms for the delivery of anti-cancer drugs, such as Doxil, Caelyx, and Myocet (for doxorubicin delivery); DaunoXome (for daunorubicin delivery); Mepact (for mifamurtide delivery); and NanoTherm (for Fe_2_O_3_ delivery) (Rodríguez et al., 2022); infers a promising future for the NP-driven delivery of miRNA-based medicines.

Some of the major nanocarriers employed in miRNA-based cancer therapeutics are liposomes, exosomes, dendrimers, mesoporous silica NPs (MSN), quantum dots, gold NPs (AuNPs), iron oxide NPs (IONPs), core-shell nanomaterials, among others (Boca et al., 2020). These nanotechnological devices provide diverse advantages when harnessed as delivery systems for miRNA mimics and antagomirs. For example, ncRNAs encapsulated in cationic NPs (especially cationic liposomes) tend to show increased uptake from target cells due to the fact that such NPs easily interact with the negatively charged surface of the cell membrane (Silva-Cazares et al., 2020; Sukocheva et al., 2022); otherwise, the negative charge and molecular weight of miRNAs would hinder their passage across the cell membrane (Segal and Slack, 2020). Cell uptake of the nanocarrier can also be facilitated by coating it with tumor-specific targeting ligands, which in turn can reduce undesired off-target effects of miRNA mimics or antagomirs on healthy cells (Amaldoss et al., 2022; Kara et al., 2022).

**FIGURE 2:**
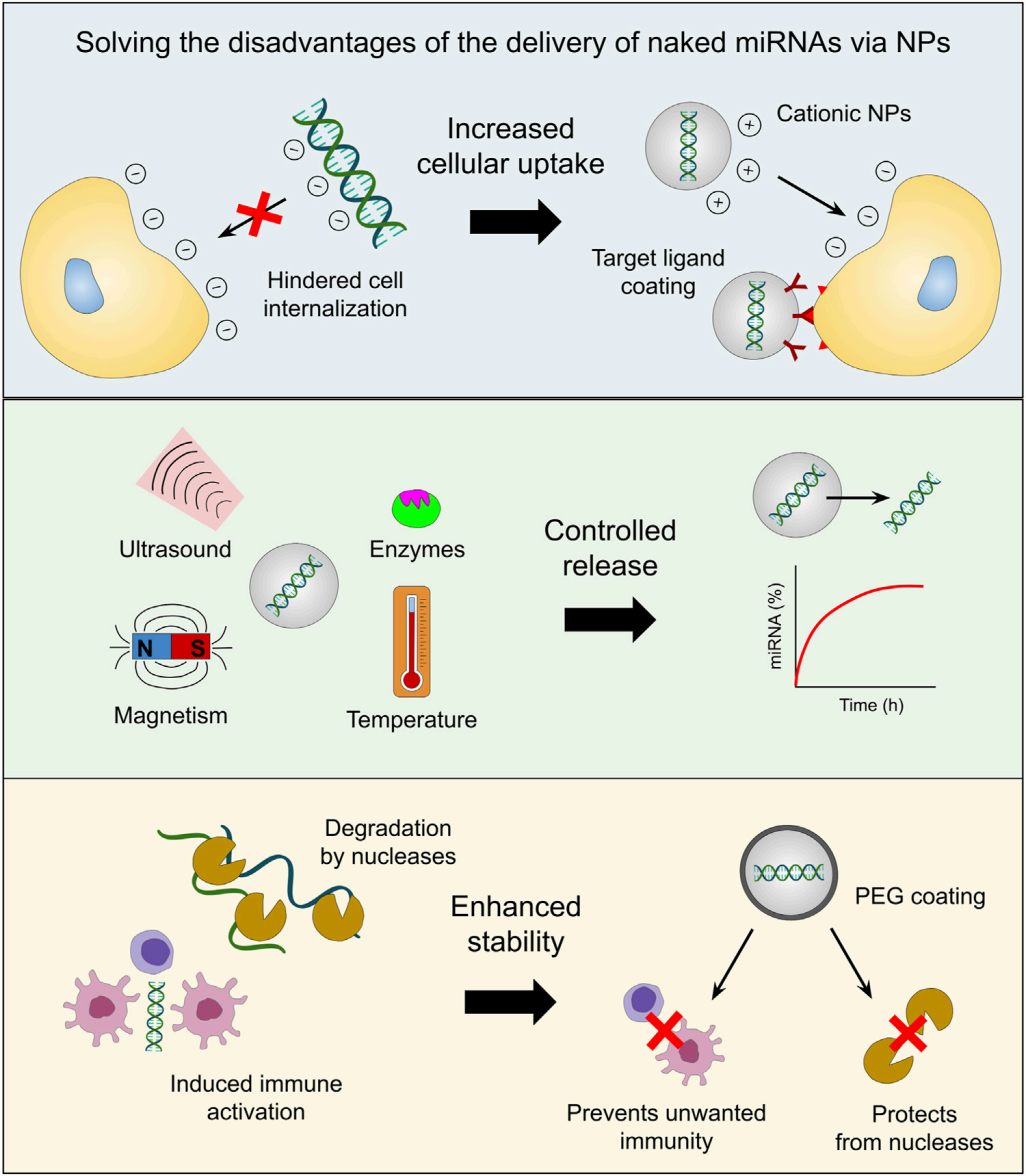
Advantages of the NP-mediated delivery of miRNA-based drugs. Nucleic acids, as well as the cell membrane, are negatively charged, which makes cell internalization of naked miRNAs a challenge. Cationic NPs envelop miRNAs in a positively-charged shield which improves cellular uptake. Moreover, the addition of target ligands in the outer layer of NPs can enhance selectivity and reduce off-target effects. Furthermore, controlled release of the therapeutic cargo can be achieved when the NPs are in contact with stimuli such as specific enzymes, ultrasound, magnetic fields and temperature, to name a few. Additionally, nanoencapsulation boosts miRNA stability by protecting them from nuclease degradation and inhibiting undesired immune activation through the implementation of PEG-based coating.

Another advantage of cancer nanomedicine consists of the fact that drugs can be released in a controlled manner under specific stimuli, such as pH, redox potential, temperature, reactive oxygen species (ROS), hypoxia conditions, ultrasound, magnetic fields, electrical fields, the light at different wavelengths (e.g., ultraviolet, visible, near-infrared), or under the presence of specific enzymes (Jain et al., 2020; Moradi Kashkooli et al., 2020). Nanocarriers also protect artificial miRNAs from degradation by nucleases and increase their stability, which makes it possible to deliver appropriate doses of these molecules to achieve the desired clinical effect in cancer cells (Desantis et al., 2020; Asakiya et al., 2022). On the other hand, since artificial miRNAs can induce immune responses characterized by the secretion of inflammatory cytokines and type I interferons (IFNs), miRNA mimics and inhibitors delivered through nanotechnological platforms are less likely to trigger the unfavorable immunogenic activity of tumor-associated immune cells (e.g., macrophages and monocytes) (Mollaei et al., 2019; Forterre et al., 2020). In fact, it has been proved that the presence of a stealth coating (like polyethylene glycol [PEG]) covering the surface of NPs prevents immune cell activation (Roy et al., 2022). As a result, these advantages (illustrated in Figure 2) give hope that, in the following years, there will be a significant trend toward the creation, improvement, pre-clinical and clinical testing of protocols for the nanotechnology-mediated delivery of miRNA-drugs.

## 3 Emerging applications of nanotechnologies for miRNA-mediated cancer therapeutics

### 3.1 Breast cancer

Breast cancer is a heterogeneous disease distinguished by the growth of malignant tumors in mammary glands (Heer et al., 2020). Clinical particularities of this cancer comprise breast lumps, armpit/breast pain, breast skin redness, nipple bleeding, nipple rash, change in the morphology of the nipple, weight loss, and generalized fatigue, among other symptoms (Elshami et al., 2022). Unfortunately, this disease represents the most common cancer with over 2.3 million new cases worldwide and 685,000 deaths in 2020, which accounted for 16% of cancer deaths in women (Arnold et al., 2022). Given the elevated prevalence and mortality rates of breast cancer, novel genetic therapies are currently under development, intending to improve patients’ quality of life. Specifically, miRNA-loaded NPs have shown to be a promising treatment as they can precisely target and silence oncogenes and multiple breast-carcinogenic-related pathways.

**FIGURE 3:**
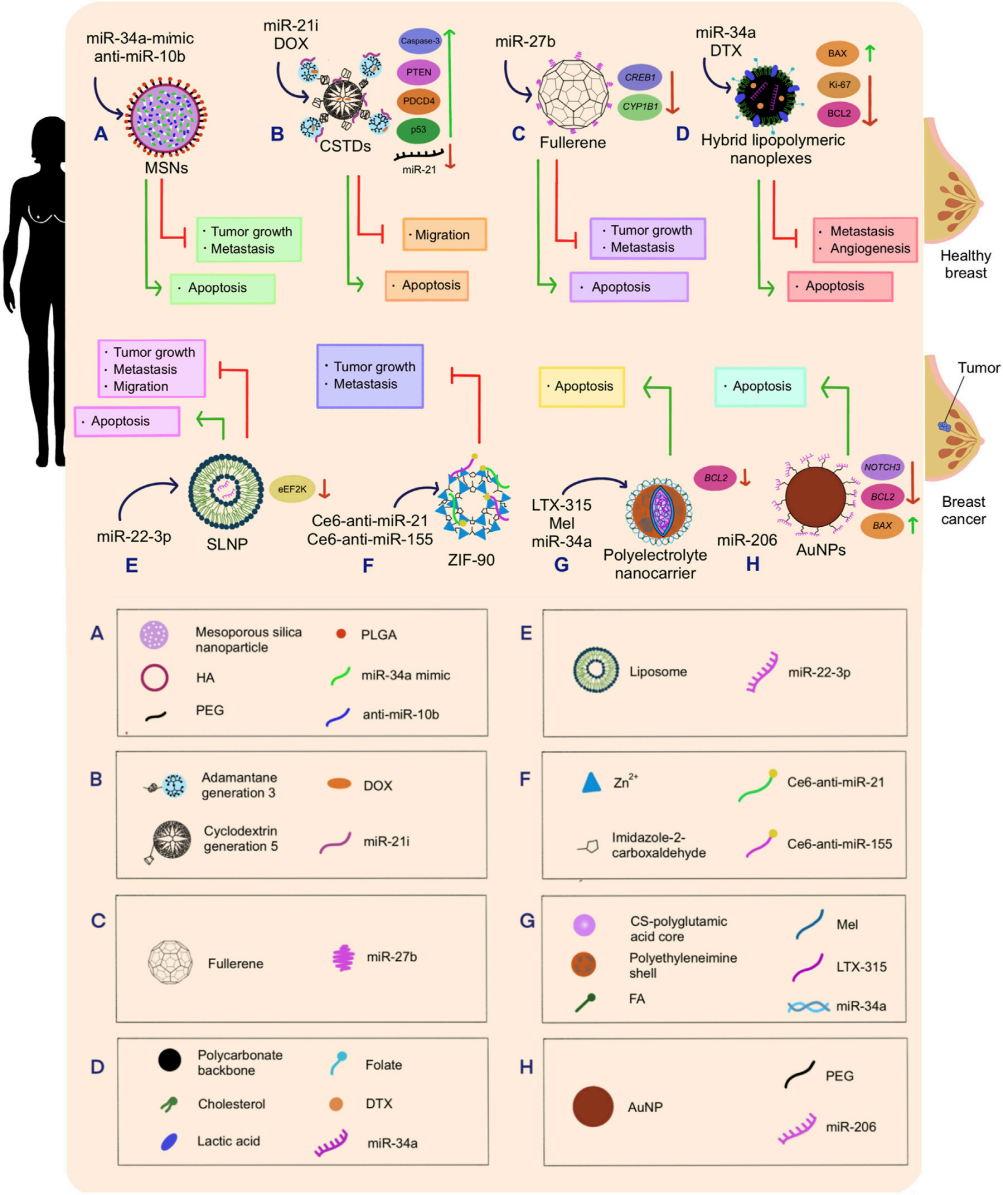
Schematic representation of the therapeutic effects of nanotechnology-mediated miRNA therapeutics on breast cancer. **(A)** miR-34a-mimic and anti-miR-10b were co-delivered into breast cancer cells using MSNs as delivery vehicles, triggering tumor growth inhibition and metastasis delay. **(B)** miR-21 inhibitor and DOX drug were co-delivered into breast tumors using CSTDs built with a generation 3 adamantane and a generation 5 cyclodextrin, which caused anti-migratory effect, and apoptosis of breast cancer cells. Additionally, this therapy produced the upregulation of Caspase-3, PTEN, PDCD4, and p53; and the downregulation of miR-21. **(C)** miR27b was conjugated with fullerenes and released into PTX-resistant breast cancer cells, which allowed the regulation of tumor growth, suppression of metastasis, and enhanced apoptosis. Further, this treatment downregulated *CREB1* and *CYP1B1* expression. **(D)** miR-34a and DTX were delivered into breast cancer cells employing hybrid lipopolymeric nanoplexes mainly composed by a polycarbonate backbone coupled with cholesterol, lactic acid, and folate. This treatment induced the inhibition metastasis and angiogenesis, increased apoptosis, caused the downregulation of Ki-67 and BCL2, and upregulation of BAX. **(E)** miR-22-3p transfected into breast tumors through SLNP inhibited tumor growth, metastasis, and migration. Moreover, eEF2K became downregulated. **(F)** Ce6-anti-miR-21 and Ce6-anti-miR-155 were co-delivered into breast cancer cell lines employing ZIF-90, a nanoparticle constructed with zinc ions and imidazole-2-carboxaldehyde. Consequently, tumor growth and metastasis were reduced. **(G)** LTX-315, Mel, and miR-34a were synthesized with a polyelectrolyte nanocarrier composed of a CS-polyglutamic acid core protected by a polyethyleneimine shell and FA ligands. This combination of molecules caused the targeted breast cancerous cells to undergo apoptosis. **(H)** miR-206 was loaded into PEG-conjugated AuNPs, and transported to breast cancer cells, triggering apoptosis, downregulating *NOTCH3, BCL2*, and upregulating *BAX*.

In such regard, miR-21, responsible for promoting tumor growth and metastasis, has been reported to be overexpressed in triple-negative breast cancer (TNBC) tumors, as well as in other types of cancer (e.g., lung cancer and glioblastoma). In fact, miR-21 is a well-established oncomiR. On this basis, a miR-21 inhibitor (miR-21i) was conjugated with core-shell tecto dendrimers (CSTDs) to target this RNA molecule in TNBC cell lines (Figure 3B). Moreover, previous work has shown that the co-delivery of chemotherapeutic drugs along with miRNA inhibitors enhances cytotoxic response compared to the drug alone. Thus, the anticancer drug doxorubicin (DOX) was encapsulated inside the CSTDs/miR-21i polyplex for co-delivery. Polyplexes (polymeric systems electrostatically bound to nucleic acids) were successfully internalized by MDA-MB-231 human breast adenocarcinoma cells (Song et al., 2020). Furthermore, efficient *in vitro* transfection of miR-21i by the polyplexes induced apoptosis activation and strong anti-migratory effect in the treated cancer cells due to the downregulation of miR-21 expression and significant upregulation of miR-21 targets involved in the regulation of tumor proliferation, such as phosphatase and tensin homolog (PTEN), programmed cell death protein 4 (PDCD4), tumor protein p53 (p53), and caspase-3. Overall, this study highlighted the applicability of CSTDs for the synergistic administration of miRNAs and chemotherapeutic drugs for improved antitumor effects (Song et al., 2020).

Ahir et al. (2020) designed MSNs to deliver miR-34a-mimic and antisense-miR-10b as a novel therapeutic approach that could retard tumor metastasis in TNBC since miR-10b is upregulated in this type of cancer, while miR-34a is downregulated. This abnormal miRNA expression encourages tumor formation and dissemination. On that account, simultaneous overexpression of miR-34a and downregulation of miR-10b was pursued. A cationic basic side chain was attached to the NPs’ core to enhance the MSN interaction with cell membranes, providing it with the capability of transporting dual miRNAs with amplified efficiency. Moreover, the loaded MSNs were coated with a hyaluronic acid-poly (ethylene glycol)-poly (lactide-co-glycolide) polymer (HA-PEG-PLGA), as it provides protection against degradation and better affinity with the CD44 receptor (overexpressed in TNBC cells). Finally, the results of this therapeutic method yielded effective inhibition of tumor growth, cancer cell death, and delayed metastasis in TNBC *in vitro* and *in vivo* (Ahir et al., 2020) (Figure 3A).

The expression of miR-22-3p has been reported to be downregulated in TNBC cells. This miRNA regulates the eukaryotic elongation factor 2 kinase (eEF2K), which promotes the proliferation and invasion of cancer cells, enhances tumor formation, and triggers drug resistance when overexpressed. Thus, increased miR-22-3p levels are essential to inhibit eEF2K carcinogenic functions. Gorur et al. (2021) transfected MDA-MB-231 and MDA-MB-436 cell lines with miR-22-3p. Later, grafts of the same type of cell lines were put into the mammary fat of mice, which were then injected with single-lipid liposomal NPs (SLNPs) loaded with miR-22-3p. In both cases, expression levels of miR-22-3p were restored, silencing eEF2K, inducing apoptosis, and inhibiting cancer cell proliferation, colony formation, invasion, and metastasis (Gorur et al., 2021) (Figure 3E).

Subsequently, a targeted multilayer FA-decorated polyelectrolyte nanocarrier was designed by Motiei et al. (2021) to deliver LTX-315 (an oncolytic peptide with antitumor activity) and Melittin (Mel) peptides, as well as miR-34a into the MDA-MB-231 breast cancer cell line. MiR-34a, involved in tumor proliferation, invasion and metastasis, is commonly underexpressed in this type of cancer. Meanwhile, LTX-315 is a peptide that has been identified to trigger immune responses against cancer by causing mitophagy. On the other hand, Mel is a 26 amino acid peptide capable of downregulating B-cell lymphoma 2 (*BCL2*) antiapoptotic protein and upregulating p53 proapoptotic protein. Additionally, Mel can downregulate the expression of cell division-regulating genes cyclin D1 and *CDK4*, thereby inhibiting cancer cell proliferation (Motiei et al., 2021). On the whole, *BCL2* and *cyclin D1* gene downregulation, as well as low *caspase-8* activity (triggered by the synergistic regulatory activity of miR-34a and Mel) intrinsically induced apoptosis and inhibited cell proliferation. Nonetheless, *caspase-8* showed enhanced activity under the presence of LTX-315, suggesting apoptosis induction via the extrinsic way (Motiei et al., 2021) (Figure 3G).

It has been well established that miR-34a is downregulated in breast cancer and plays a key role in regulating genes involved in the cell cycle, such as cyclin-dependent kinase 4 (*CDK4*) and *CDK6*, as well as *BCL2*, which participates in apoptotic mechanisms. On the other hand, docetaxel (DTX) is a chemotherapeutic drug that promotes cell cycle arrest, impaired mitosis, and apoptosis. However, this agent possesses low solubility in water, a fast body clearance rate, non-specific biodistribution, and can generate drug resistance. In order to maximize the stability and the antitumoral effects of both miR-34a and DXT, they were co-delivered into 4T1 and MDA-MB-231 cell lines using folate-targeted hybrid lipo-polymeric nanoplexes composed mainly by a polycarbonate backbone and cholesterol (Sharma et al., 2021). The folate-targeted polycarbonate backbone from the nanoplexes facilitated endo-lysosomal fate escape, avoiding payload degradation. Besides, the folate moiety enhanced active cancer cell targeting due to the overexpressed folate receptors located on the surface of carcinogenic cells. Finally, the cholesterol grafted to the backbone allowed lipid-mediated endocytosis (Sharma et al., 2021).

This delivery method demonstrated an optimal encapsulation and delivery of the two therapeutic molecules of interest since BCL2 anti-apoptotic protein was found to be downregulated after the treatment. In this case, miR-34a worked in synergy with DTX to reduce *BCL2* expression since the miRNA avoided protein synthesis via gene silencing, while the drug inhibited BCL2 phosphorylation (Sharma et al., 2021). Once the treatment was completed, BCL2-associated X (BAX) pro-apoptotic protein expression was enhanced, and Ki-67 antigen nuclear protein (responsible for tumor cell proliferation) was downregulated. Further, inhibition of angiogenesis, proliferation, metastasis, and cancer progression were detected, in addition to epithelial-mesenchymal transition (EMT) regression and enhanced apoptosis (Sharma et al., 2021) (Figure 3D).

In another recent study, antisense oligonucleotides (anti-miRNAs) were designed against the metastasis-associated and chemoresistance-associated miRNAs miR-155 and miR-21. To improve anti-miRNAs’ effectiveness, photodynamic therapy was also incorporated into the system at issue by adhering the chlorin E6 (Ce6) photosensitizer to anti-miR-21 and anti-miR-155 (Shang et al., 2022). In fact, Ce6 reacts to 660 nm wavelengths, resulting in energy liberation, which in turn reacts with oxygen molecules to produce reactive oxygen species (ROS), triggering oxidative stress that eventually leads to apoptosis. Importantly, superhydrophobic zeolitic imidazolate framework NP (ZIF-90) was employed to deliver Ce6-anti-miR-21 and Ce6-anti-miR-155 into human breast cancer cell line MDA-MB-231. The pH responsiveness of ZIF-90 allowed it to self-decompose and release its nucleic acid content upon exposure to the acidic environment in tumor cells. Consequently, it was found that the combination of photodynamic therapy and anti-miRNA-controlled release suppresses metastasis and tumor cell proliferation (Shang et al., 2022) (Figure 3F).

Proper conjugation of AuNPs with miRNAs represents a challenging drawback in the field of NP-mediated miRNA delivery. Nonetheless, said issue was overcome by Chaudhari et al. (2022), who developed a simpler method for the fabrication of miRNA-loaded AuNPs with the aim of treating luminal-A type of breast cancer with miR-206, which has been reported to be downregulated in this type of breast cancer and involved in metastasis. These authors developed a complex consisting of AuNPs with limited toxicity inferred by the citrate capping method, further modified with a polyethylene glycol (PEG) moiety to provide a positive charge that simplifies miRNA attachment and stabilizes the platform. After several *in vitro* assays, it was evidenced that the engineered system reduced the cell viability of MCF-7 breast cancer cells, induced G0-G1 cell arrest, and activated mitochondria-mediated apoptosis. Remarkably, even under nanomolar (nM) concentrations of the miR-206, Au-PEG-miRNA NPs triggered cancer cell death by hindering the expression of a well-known target of miR-206, neurogenic locus notch receptor 3 (*NOTCH3*, responsible for cell proliferation and tumorigenesis). Furthermore, the nanoplatform downregulated *BCL2* and increased *BAX* expression (Chaudhari et al., 2022) (Figure 3H).

Current research has shown that the downregulation of miR-27b is linked with chemoresistance. Under that premise, expression induction of this miRNA was carried out with the purpose of reversing paclitaxel (PTX) resistance in breast cancer cell lines (MCF-7/Adr) and breast cancer-bearing mice. To carry out this experiment, single-stranded miR-27b was conjugated with fullerene C_60_ (also known as buckminsterfullerene) to synthesize a fullerene nanospherical miRNA. This nanocarrier was then phagocytized by the cells, where miR-27b is being released (Xu et al., 2022). Subsequently, this miRNA triggered the downregulation of cyclic adenosine monophosphate responsive element binding protein 1 (*CREB1*) and cytochrome P450 family 1 subfamily B member 1 (*CYP1B1*) proteins at the transcriptional level. CREB1 regulates the proliferation, survival, and differentiation of cancerous cells, while the latter is involved in drug detoxification. On the other hand, fullerene nanospheres stimulated ROS production under 365 nm irradiation. Altogether, ROS output, along with *CREB1* and *CYP1B1* downregulation, reverted PTX recalcitrance, allowing apoptosis and suppression of metastasis in resistant breast cancer cells in both *in vivo* and *in vitro* assays (Xu et al., 2022) (Figure 3C).

Although promising, the current studies investigating the delivery of miRNAs via NPs for the treatment of breast cancer have certain limitations that need to be addressed. One of the major concerns in this matter corresponds to the unknown adverse effects of the nanocarriers employed in the delivery of miRNA-based drugs. A comprehensive safety profile evaluation is necessary to assess potential side effects and mitigate any risks before advancing to clinical trials. As well, understanding the biodistribution and potential accumulation of NPs in various organs and tissues is essential for assessing their systemic impact and ensuring patient safety.

Besides, determining the optimal dosage, frequency of administration, and duration of treatment is crucial for achieving maximum therapeutic efficacy while minimizing off-target effects. Accordingly, the interactions between miRNAs and NP, their pharmacokinetics, pharmacodynamics, and specific mechanisms of action in breast cancer cells need to be elucidated. More importantly, conducting well-designed pre-clinical and clinical trials is essential to validate the safety, efficacy, and overall therapeutic potential of miRNA-loaded NPs in breast cancer.

### 3.2 Lung cancer

Lung cancer is characterized by the presence of thymic, lymph-node, vascular, connective, or neurogenic tumors in the lung, pleura, or mediastinum, which are caused by mutations that commonly affect epidermal growth factor receptor (*EGRF*), kristen rat sarcoma viral oncogene (*KRAS*), phosphatidylinositol-4,5-biphosphate 3-kinase catalytic subunit alpha (PIK3A), and *PTEN* (Bironzo et al., 2022). Although the estimated heritability of these mutations is 18% (Rifkin et al., 2023), most lung cancer cases are triggered by smoking (Rustagi et al., 2023). Individuals affected by this disease present a cluster of symptoms encompassed by chest pain, fatigue, sleep deprivation, cough, dyspnea, hemoptysis, and weight loss (Ruano-Raviña et al., 2020; Luo et al., 2023). Thereupon, given the escalated incidence and severity of this type of cancer, miRNA-loaded NPs have received significant attention in the last years due to their clinical significance in the modulation of oncogenic protein expression.

**FIGURE 4:**
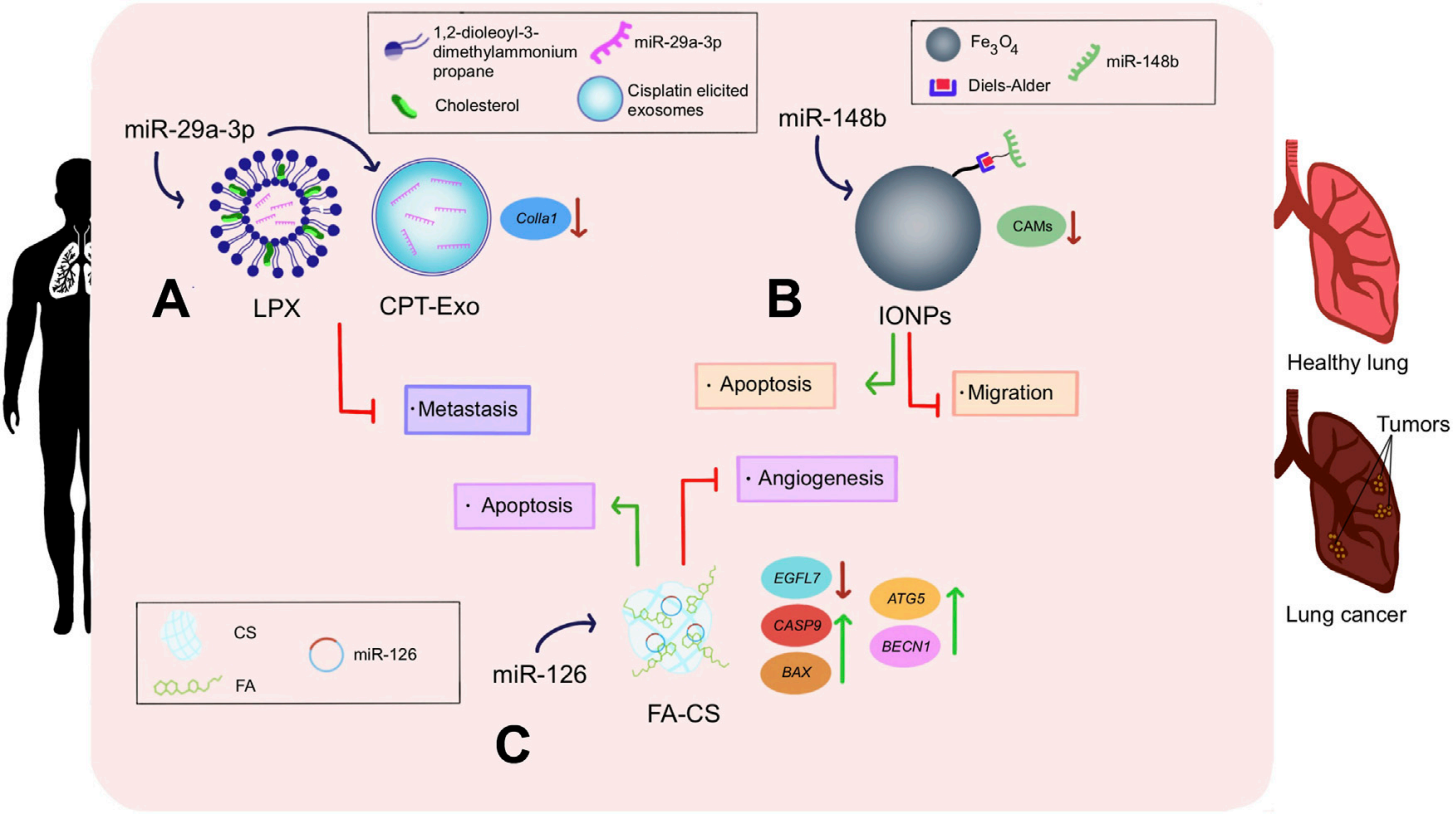
Impact of nanotechnology-mediated miRNA therapeutics in lung cancer. **(A)** miR-29a-3p was delivered into lung cancer cells employing LPX and CPT-Exo. DOTAP was conjugated with cholesterol to surround miR-29a-3p in the LPX nanoparticle. Meanwhile, CPT-Exo nanoconjugate consisted of miR-29a-3p protected by cisplatin-elicited exosomes. In both cases, decreased metastasis and downregulation of *Colla1* were noticed. **(B)** miR-126 was liberated into lung cancerous cells using FA-CS as a nano vehicle. This triggered apoptosis of damaged cells, downregulation of *EGFL7*, *CASP9*, and *BAX*; as well as upregulation of *ATG5* and *BECN1*. **(C)** miR-148b was chemically attached to iron oxide nanoparticles through the Diels–Alder linker, and delivered into lung tumors, resulting in apoptosis, suppression of CAMs, and a decrease of cancerous cell migration.

As a matter of fact, the lungs are one of the most common sites for cancer metastasis since the presence of collagen in this organ facilitates the dissemination of tumor cells. Yan et al. (2022b) studied for the first time the therapeutic efficacy of miR-29 on inhibiting collagen expression in the lung. Data analysis showed that miR-29a-3p negatively regulates the expression of collagen I (*Col1a1*), and the upregulated expression of said miRNA is associated with better prognosis in patients. Therefore, in order to deliver miR-29 into mouse lung fibroblasts, they designed two nanovesicle systems. The first one consisted of naturally elicited exosomes upon cisplatin chemotherapeutic treatment (CPT-Exo), which successfully downregulated *Col1a1* both *in vitro* and *in vivo*, therefore decreasing tumor cell metastasis. The second one comprised a liposome-based nano-delivery system referred to as lipoplex (LPX), which turned out to be less biocompatible than CPT-Exo as they are easily cleared by immune cells. Nonetheless, LPX had enriched tissue-specific delivery attributed to the previously optimized (Kranz et al., 2016) charge ratio adjustment of 1,2-dioleoyl-3-trimethylammonium propane (DOTAP)/cholesterol to negatively-charged miRNA (4:1). This proportion facilitated the targeted delivery of the miR-29a-3p-LPX nanosystem specifically to lung tissues. Even so, in both cases, the ability shown by the efficient delivery of miR-29a-3p alleviated the establishment of a pro-metastatic environment for circulating lung tumor cells (Yan et al., 2022b) (Figure 4A).

Notably, folic acid (FA) receptors are widely overexpressed on the surface of cancerous lung cells. Accordingly, FA can be used as a targeting ligand that would covalently bind to these receptors and induce endocytosis of the conjugated drug delivery material, ultimately enhancing cellular uptake. On the other hand, chitosan (CS) is a highly abundant polysaccharide with several advantages as a drug delivery material, including non-toxicity, non-immunogenicity, stability, and the fact that its amino-functional surface groups can be easily modified and functionalized. In this context, Golafzani et al. (2022) designed an FA-decorated CS (FA-CS) NP platform to effectively deliver miR-126 to A549 lung cancer cells. Quantitative Real-Time PCR (qRT-PCR) revealed the improved ability of miR-126 delivered through the FA-CS nano-complex to downregulate the expression of epidermal growth factor-like domain 7 (*EGFL7*), an angiogenic promoter. Besides, an increased expression of the apoptosis inductors *caspase-9* and *BAX* genes was observed. As well, miR-126 enhanced the expression of genes with an important role in autophagy induction, such as autophagy-related 5 (*ATG5*) and beclin 1 (*BECN1*). Altogether, the FA-CS-miR-126 delivery system demonstrated cancer inhibition *in vitro* (Golafzani et al., 2022) (Figure 4C).

MiR-148b has been reported to suppress a class of proteins in non-small cell lung cancer (NSCLC) called cell adhesion molecules (CAMs), which are responsible for intercellular attachment, adhesion to surfaces, and cell migration. In fact, expression levels of miR-148b have been found to be inversely proportional to A549 NSCLC cells’ survivability. In this regard, Arrizabalaga et al. (2022) designed IONPs with chemical attachment sites on their surface as ideal spots for miRNA junctions. It is noteworthy to emphasize that the oligonucleotide was coupled with the NP using a Diels–Alder linker, which permitted the spatiotemporally controlled miRNA delivery into Human Caucasian Lung Carcinoma cells (A549) when stimulated by alternating magnetic field radiofrequency (AMR-RF). Confocal microscopy demonstrated the efficient and selective uptake of the nano-complex by A549 cells. Meanwhile, viability assays confirmed the subsequent induction of cytotoxicity, with significantly enhanced results in the groups that included AMR-RF compared to those in which AMR-RF was not applied. Viability tests also evidenced that AMR-RF-exposed A549 cells displayed less survival rates (Arrizabalaga et al., 2022) (Figure 4B).

According to the results of the studies herein, the delivery of miRNAs via NPs holds promise as a potential treatment strategy for lung cancer. However, several constraints persist that hinder their successful translation into clinical practice. One key limitation is the lack of knowledge regarding the adverse effects of NP-mediated miRNA delivery in lung cancer patients. While *in vitro* and animal studies have shown positive outcomes, the long-term safety and potential side effects in humans remain uncertain. Hence, thorough evaluation and monitoring of potential toxicities are essential to ensure patient safety and effectively mitigate any risks before progressing to human trials.

Moreover, the specific biological and physiological characteristics of lung cancer pose additional challenges in the nanocarrier-mediated delivery of miRNA-focused drugs. In this sense, the complex tumor microenvironment, heterogeneous cell populations, and variations in gene expression patterns necessitate a deeper understanding of the pharmacodynamics and pharmacokinetics of these nanoformulations. To overcome these limitations, it is crucial to employ appropriate biological models that closely mimic the specific conditions of lung cancer in humans (e.g., patient-derived xenografts, lung cancer organoids, and 3D cell cultures). By utilizing relevant models that faithfully replicate the characteristics of lung tumors and the surrounding microenvironment, researchers could obtain more accurate insights into treatment responses, potential adverse effects, and overall efficacy, thereby improving the chances of successful clinical translation.

### 3.3 Glioblastoma

Glioblastoma is a type of cancer characterized by glioma development within the central nervous system, particularly in the brain (Tołpa et al., 2023). This type of brain malignancy represents the most lethal and aggressive primary neoplasm, given that it has a median survival of 14 months and only 5% of the patients survive beyond 5 years after being diagnosed (Zhong et al., 2020; Huan et al., 2023). Clinical indicators of glioblastoma commonly include headaches, seizures, nausea, vomiting, alterations in personality, imbalance when walking, urinary incontinence, and visual difficulties (Hansen et al., 2023). Additionally, the prognosis remains unfavorable despite the remarkable advances in neuro-oncology and biomedicine. In the last years, nanomedicine has shown important potential for NP-mediated local targeted delivery of miRNAs, which are able to simultaneously regulate mutated tumor genes that promote proliferation, differentiation, and motility in glioblastoma.

**FIGURE 5:**
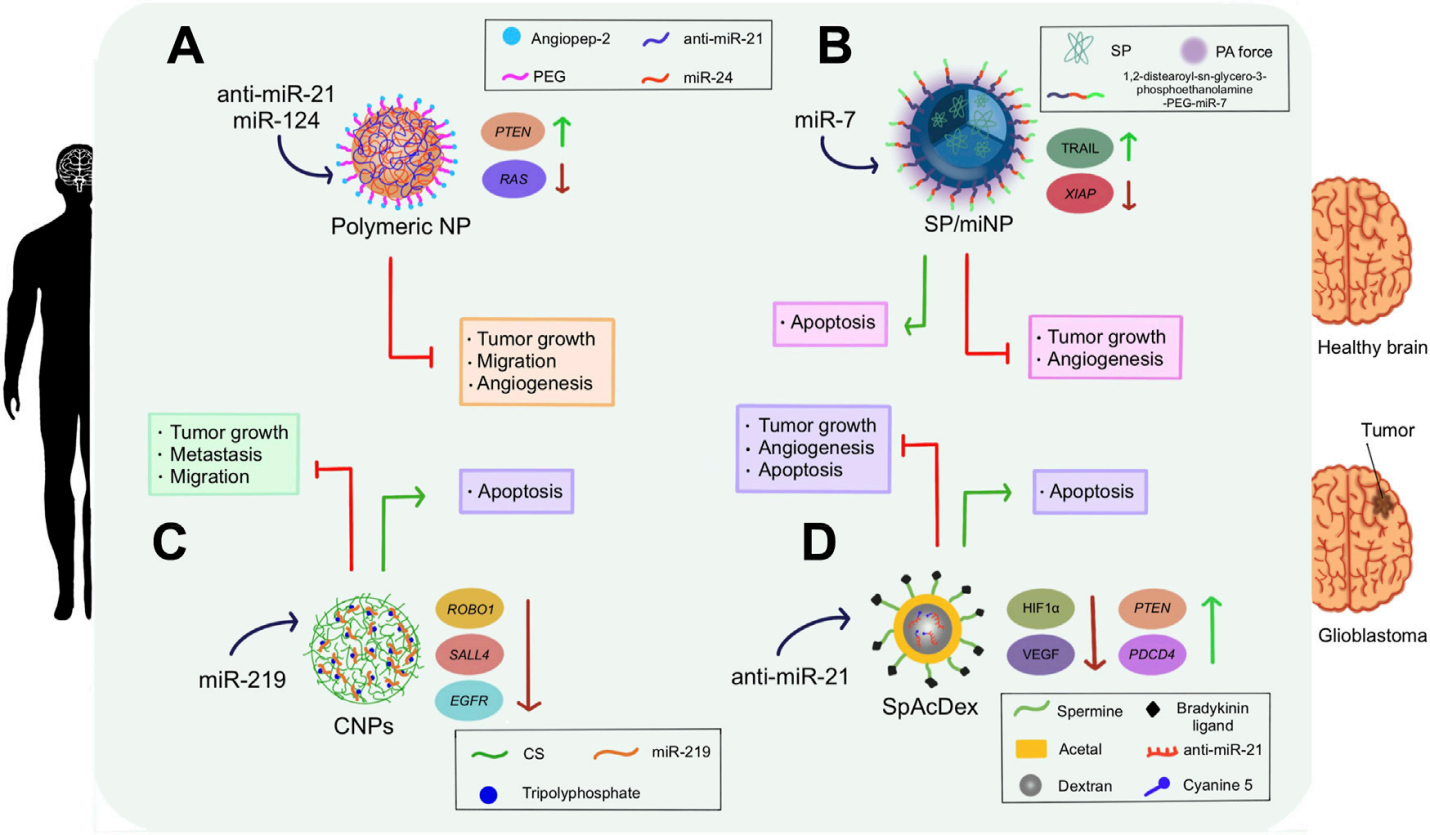
Clinical inferences of NP-delivered miRNA therapeutics in glioblastoma cells. **(A)** anti-miR-21 and miR-124 were administered into cancerous brain cells using polymeric NPs NPs coated with angiopep-2 and PEG. These miRNAs reduced cell proliferation, migration, and angiogenesis; and promoted apoptosis of brain cancer cells. Furthermore, they increased PTEN expression while decreasing RAS expression. **(B)** A semi-conducting polymer decorated with 1,2-distearoyl-sn-glycero-3-phosphoethanolamine and PEG liberated miR-7 inside brain tumors under PA force stimuli. This delivery triggered a reduction in tumor growth and angiogenesis. Also, it caused the apoptosis of the cancerous cells, upregulated TRAIL, and lowered the expression of *XIAP*. **(C)** miR-219 was loaded into CNPs with tripolyphosphate and transferred into glioblastoma cells. This therapy decreased metastasis, promoted apoptosis, and reduced cell migration and proliferation. *ROBO1, SALL4*, and *EGF*R were downregulated after this treatment. **(D)** anti-miR-21 was loaded onto the SpAcDex nanoparticle system and delivered into brain tumors, inhibiting angiogenesis and tumor growth; and stimulating apoptosis. Besides, it downregulated HIFα and VEGF, while upregulating *PTEN* and *PDCD4*. The SpAcDex nanostructure consisted of a dextran core enveloped by an acetal layer and decorated by a spermine and bradykinin ligand conjugate. Cyanine 5 served as a fluorophore marker to indicate the presence of anti-miR-21.

In an intriguing investigation, a polymeric NP was developed to deliver anti-miR-21 and miR-124 into glioblastoma multiforme (GBM) tissue. Interestingly, oligopeptide angiopep-2 was incorporated to ensure specific binding of the NP to the highly expressed lipoprotein receptor-related protein 1 (LRP-1) receptors in GBM tumor cells. This promoted enhanced GBM uptake and easy traverse of the blood-brain barrier. Moreover, angiopep-2 was able to encapsulate and protect aforesaid miRNAs from degradation via electrostatic, hydrogen-bond, and hydrophobic interactions. Significantly, these interactions became neutralized when the NP encountered the ROS stimuli present in tumor cells, which enabled precise miRNA release (Liu et al., 2021). The delivery of anti-miR-21 via polymeric NPs repressed the expression of miR-21, permitting the tumor suppression activity of PTEN. On the other hand, miR-124 has been found to be downregulated in GBM; this miRNA is capable of inhibiting tumor growth and invasion by targeting the rat sarcoma (*RAS*) family, which is responsible for controlling cell division (Liu et al., 2021). Consequently, the delivery of miR-124 into cancerous brain cells provoked tumor growth deceleration. On the whole, anti-miR-21 and miR-124 synergic activity in orthotopic GBM xenograft models demonstrated a strong antitumor effect since diminished proliferation, invasion, angiogenesis, and migration of tumor cells was detected, leading to higher survival rates (Liu et al., 2021) (Figure 5A).

It has been elucidated that miR-7 is able to sensitize TRAIL to effectively repress tumors. Likewise, the X-linked inhibitor of apoptosis (*XIAP*) is a target gene of miR-7 and is responsible for the regulation of cell death sensitivity (Kang et al., 2021b). In such a regard, miR-7 was loaded into a near-infrared (NIR) absorbing semiconducting polymer-based/miRNA nanocarrier (SP/miNP). The primary advantage that this NP possess relies on the fact that it is capable of generating strong photoacoustic radiation (PA) force when a NIR-pulse laser is applied toward the target tissues and vessels. The SP then absorbs light, allowing it to smoothly overcome the endothelial barriers so that rapid and intact delivery of miR-7 into U87-GM-bearing mice could be pursued. Furthermore, this gene therapy was improved with a TRAIL-inducing compound (TIC), which worked synergistically with miR-7 to trigger TRAIL-induced apoptosis. Assays demonstrated that this treatment achieved miR-7 intact delivery in 20 min, resulting in the inhibition of tumor growth, apoptosis induction, and angiogenesis reduction by downregulating the expression of XIAP and upregulating TRAIL expression (Kang et al., 2021b) (Figure 5B).

Later, anti-miR-21 was loaded onto a spermine-modified acetalated dextran NP system (SpAcDex), whose highly positive charges allowed a miRNA encapsulation efficiency of 90% (Zheng et al., 2022). Additionally, the nanomaterial was conjugated with bradykinin peptide, which exhibits great affinity for the B1 receptor, highly expressed in brain tumor microvascular systems. When the association between the bradykinin ligand and its B1 receptor counterpart is established, the permeability of vessels in the blood-tumor barrier (BTB) is adjusted, facilitating SpAcDex penetration and uptake by GBM tumor cells, allowing an efficient cargo release. *In vivo* assays performed in orthotopic U87MG glioma-bearing mice, as well as *in vitro* trials in human (U87MG) and rat (C6) glioma cells displayed continuous anti-miR-21 release, cell apoptosis, tumor growth deceleration, and blood vessel normalization (Zheng et al., 2022) (Figure 5D).

MiR-219 is known to be downregulated in GBM. In fact, data indicates that miR-219 overexpression triggers the downregulation of both roundabout guidance receptor 1 (*ROBO1*) and spalt-like transcription factor 4 (*SALL4*), which are linked with prolonged patient survival. To investigate their gene delivery potential, chitosan NPs (CNPs) loaded with miR-219 were constructed using an ionic gelation method, achieving an entrapment efficiency of 95% (Alswailem et al., 2022). Subsequently, sustained release patterns of this miRNA from the complex reduced tumor proliferation, migration, and invasion of U87 MG human brain cancer cells without affecting fibroblasts *in vitro* (representing healthy cells), which suggests that this NP-mediated treatment is safe and specific. Said effects were proved to be mediated by the downregulation of miR-219 target genes epidermal growth factor receptor (*EGFR*)*, ROBO1* and *SALL4,* inducing apoptosis (Alswailem et al., 2022) (Figure 5C).

In spite of the fact that some reports have shown encouraging outcomes, the use of miRNA nanoformulations for glioblastoma treatment still faces several challenges that need to be addressed. For instance, the current models used in glioblastoma research do not fully mimic the complexity of human disease. Traditional cell culture models or animal models may not adequately replicate the specific microenvironment of glioblastoma, including the interactions with surrounding cells and the blood-brain barrier. Therefore, it is essential to develop advanced biological models that better capture the unique characteristics of glioblastoma, such as patient-derived xenografts or three-dimensional organoids, to improve the translatability of miRNA nanoformulations into clinical trials.

On the other hand, the lack of comprehensive long-term studies in animal models or clinical trials hinders our understanding of potential toxicities and off-target effects of these therapeutic approaches. Consequently, a thorough evaluation of the toxicity profiles, as well as the biodistribution and accumulation of NPs in healthy brain tissues, is crucial before advancing into clinical applications for glioblastoma patients. To overcome these limitations, comprehensive studies evaluating the pharmacodynamics and pharmacokinetics of miRNA nanoformulations in reliable animal models and eventually in human patients are necessary to optimize their therapeutic potential and ensure safe and effective treatment strategies for glioblastoma.

### 3.4 Liver cancer

Liver cancer is a type of carcinoma that grows in hepatic cells (Wu et al., 2023). It is the fourth leading cancer-related cause of mortality, and approximately 800,000 cases are diagnosed each year (Bakrania et al., 2023). Certain associated risk factors that could lead to liver cancer development are chronic viral hepatitis, alcohol abuse, excess body fat, and constant smoking (Arafa et al., 2023). The main clinical particularities of this cancer involve white stools, hepatitis B, hepatitis C, jaundice, considerable weight loss, vomiting, and abdominal pain (Mehmood et al., 2022). Nonetheless, symptoms are insidious at early disease stages, and most patients are diagnosed only when cancer reaches advanced levels (Jiang et al., 2023). Overall, given the elevated number of liver cancer-related deaths, incidence, and severity, chemo-gene combinatorial therapies have been devised to provide an alternative treatment against hepatic cancer (Wang et al., 2021c). Particularly, miRNAs have caught special attention for the diagnosis and therapy of liver cancer as they regulate the progression of oncogenes.

One of the key target miRNAs involved in hepatocarcinogenesis is miR-122 owing to the fact that it can suppress tumorigenic proteins in liver cells and downregulate permeable glycoproteins (P-gp), which work as channels that mediate the expulsion of drugs from the cell. Considering this, miR-122, the anticancer drug PTX, and hepatocyte targeting ligands were co-delivered into HepG2 cells using multivalent 6-way junction (6WJ) RNA NPs (Wang et al., 2021a). This nanoscale delivery system possesses a rubber-like property, which allows it to have amplified permeability and retention when entering the tumor’s vasculature, thus increasing its efficiency to target cancer cells. Similarly, conjugated hepatocyte targeting ligands function as an endocytosis-mediator receptor, providing 6WJ RNA NPs with higher tumor specificity (Wang et al., 2021a). *In vitro* and *in vivo* assessments demonstrated successful NP-tumor cell binding, cellular uptake, PTX intracellular retention, and induction of severe cytotoxicity; showing the potential synergistic effect of miR-122 and PTX to treat hepatocellular carcinoma (HCC). Importantly, expression levels of miR-122 target genes such as oncogenic disintegrin and metalloproteinase domain-containing protein 10 (*ADAM10*) and multi-drug resistance 1 (*MDR1*) were diminished, preventing tumorigenesis and drug efflux, respectively (Wang et al., 2021a). Successful drug retention, NP tumor specificity, correct biodistribution, low toxicity, as well as suppression of tumorigenic proteins contributed to the growth reduction of liver tumors (Wang et al., 2021a).

Hyperbranched polyamidoamine (PAMAM) functionalized with lactobionic acid (LA) was formulated in another investigation to selectively administer a miR-218 expressing DNA plasmid (pmiR-218) into HepG2 cell lines. Concretely, LA displays significant binding affinity towards asialoglycoprotein receptor 1 (ASGPR1), overexpressed in HCC. This provided PAMAM with enhanced biocompatibility and better cellular uptake via mediated-endocytosis activation. On the other hand, miR-218, expressed limitedly in HCC tissue, serves a critical function in inhibiting tumor proliferation and promoting apoptosis (Elfiky et al., 2021). *In vitro* and *in vivo* experiments demonstrated effective miR-218 restoration, as well as downregulation of the miR-218 target Homeobox A1 (*HOXA1*)*,* responsible for tumor progression. Consequently, diminished tumor proliferation was detected in addition to improved liver histological features (Elfiky et al., 2021).

Remarkably, N-acetylgalactosamine (GalNac)-decorated NPs represent a noteworthy approach in the realm of liver cancer research. This moiety displays a great affinity for asialoglycoprotein receptors, which are widely expressed on the surface of hepatocytes. Conjugation of the GalNac ligand to RNA strands enhances selectivity and uptake by hepatocellular carcinoma-positive cells (Debacker et al., 2020; Brown et al., 2021). Under such premise, GalNac-coated exosomes have been proved to effectively deliver miR-122 and PTX into liver tumors, avoiding the need for endosomal trapping and achieving increased cancer inhibition (Ellipilli et al., 2023). Nonetheless, the use of GalNac has not yet been widely applied in miRNA nano-complexes for liver cancer. Hence, exploring the incorporation of GalNac into NPs designed to deliver artificial miRNAs represents a promising avenue to harness the full potential of miRNA-based therapeutics. Moreover, a deeper inquiry into the oral administration of GalNac-decorated nano-complexes is encouraged, as it will represent an important advancement for use in chronically-ill patients (Debacker et al., 2020).

Notwithstanding the above, the use of miRNA-based drugs delivered via NPs in treating liver cancer is currently accompanied by several restraints that must be solved to reach the clinical panorama. For example, relying solely on cell cultures and mouse models for studying the efficacy of these treatments is inadequate. While convenient for initial screening, cell cultures fail to accurately represent the complexity and heterogeneity of liver cancer. Similarly, although mouse models provide some insights into tumor behavior, they do not fully mimic the intricate interactions between liver cancer cells and the human microenvironment. Thus, the translational potential of miRNA-based nanoformulations to clinical settings remains uncertain without rigorous evaluation in more sophisticated and relevant biological systems that better mimic the human liver cancer microenvironment.

Additionally, while short-term studies may demonstrate favorable results, these therapies’ long-term effects and potential adverse reactions require thorough investigation. As a vital organ responsible for numerous metabolic processes, the liver must carefully evaluate any potential hepatotoxicity or systemic effects resulting from miRNA NP treatments. Comprehensive long-term studies, including biodistribution analyses, monitoring of treatment response over extended periods, and assessment of potential off-target effects, are crucial to validate the safety and efficacy of miRNA-centered drugs delivered via nanocarriers in liver cancer treatment.

### 3.5 Other cancers

Former studies have confirmed that miR-451 regulates the expression of P-gp in several multi-drug resistant ovarian and gastric cancer cell lines. As previously stated, silencing P-gp expression is clinically important since this protein transports chemotherapeutics out of the cell. Taking this into account, calcium carbonate NPs coated with cancer cell membranes (designed with the aim of rising the tumor-localization capacity of the drug delivery system) were loaded with miR-451 and Adriamycin (Adr), and delivered into bladder cancer cells (Wei et al., 2020). Calcium carbonate mediated an effective miRNA binding process and proper biocompatibility, while the cancer cell membrane made possible NP retention within the tumor tissue and the overcoming of extracellular barriers. Finally, *in vivo* and *in vitro* studies showed successful delivery of miR-451, which significantly downregulated P-gp levels and maintained high intracellular levels of Adr due to the avoidance of drug efflux. Moreover, elevated expression of apoptotic genes *caspase-3* and *cytochrome-3* was found; while the level of *BCL2* apoptosis suppressor gene was lower compared to the control group, leading to mitochondria-mediated apoptosis. On the whole, the synergistic effect of Adr and miR-451 contributed to overcoming multidrug resistance in bladder cancer cells (Wei et al., 2020).

Omentum fibroblasts are known to secrete abundant exosomes, which are ideal gene delivery candidates due to their stability in blood circulation and efficacy in transporting nucleic acids. Since the majority of ovarian cancer (OC) patients undergo omentectomy, Kobayashi et al. (2020) proposed using omentum-derived exosomes to carry miR-199a-3p, a representative tumor suppressor miRNA, as a cancer replacement therapy. Remarkably, the nano-complex was successfully accumulated in cancer cells, inhibiting tumor proliferation and invasion. *In vitro* and *in vivo* experiments demonstrated an increase in miR-199a-3p expression and suppression of its direct target tyrosine-protein kinase Met (c-Met), as well as downstream target molecules, such as matrix metalloproteinase-2 (MMP2) and the phosphorylation of extracellular signal-regulated kinase (ERK). Reduced tumor burden and inhibition of metastasis were also reported in the groups treated with the nanodevice. However, more studies are required to properly determine the biodistribution of exosomes in animal models (Kobayashi et al., 2020).

Later, the attention was centered on cervical cancer treatment by synthesizing SiO_2_-polyethyleneimine NPs loaded with miR-let-7c-5p. When delivered, this miRNA was successfully transferred to epithelial carcinoma (HeLa) cells, inhibiting the development of cancer cells under shallow cytotoxicity conditions. As a result, miR-let-7c-5p/insulin-like growth factor 1 receptor/phosphatidylinositol-3 kinase/AKT serine/threonine kinase 1 (miR-let-7c-5p/IGF-1R/PI3K/AKT) and beta-catenin/snail family transcriptional repressor 2 (β-catenin/SLUG) were highlighted as potential targets against cervical cancer (Shao et al., 2020).

MiRNA miR-200c has been identified to stimulate the entrance of CD8^+^ T cells into tumors, elevate mutated cells’ sensitivity to apoptotic signals, suppress vascular formation around tumors, and downregulate programmed death-ligand 1 (PD-L1) expression, thus limiting its immunosuppressive action. More importantly, dabrafenib (Dab), a v-raf murine sarcoma viral oncogene homolog B1 (BRAF) inhibitor is a drug capable of slowing tumor growth, and its combination with miR-200c turned out to enhance cancer cells’ sensitivity to such medicine by inhibiting the expression of PD-L1 (Nguyen et al., 2021). These molecules were co-delivered into murine colon carcinoma cells MC-38 using chemokine receptor type 4 (CXCR4)-targeted polymeric NPs coated with poly-L-glutamic acid and a CXCR-4 antagonist peptide, mediating better binding with chemokine receptors, enhanced cytotoxicity and drug release, as well as content stability. *In vivo* and *in vitro* experiments showed that this cancer treatment method effectively inhibits PD-L1 expression and triggers a powerful immune response against accelerated cell differentiation. Remarkably, only a low dose of the drug was required to observe notable tumor suppression (Nguyen et al., 2021).

In another study, nona-arginine (R9) peptide labeled with ^125^I radioactive particles were combined with arginine-glycine-aspartate (RGD) short cyclic peptide, and Ce6 photosensitizer to build a self-assembling nano-complex to deliver miR-139-5p into cancer cells. This nano-complex was used in conjunction with Ce6-mediated photodynamic-radio cancer therapy to increase its therapeutic potential *in vitro* and *in vivo*. ^125^I radioactive particles produced a large number of free radicals, which caused DNA damage, thus propitiating proapoptotic p53 expression and, consequently, apoptosis, as well as tumor growth retardation, metastasis inhibition, and proliferation repression of HeLa cells (Wang et al., 2021b). On the other hand, miR-139-5p was critical for the regulation of DNA maintenance and repair genes DNA polymerase theta (*POLQ*)*,* DNA topoisomerase 1 (*TOP1*)*,* DNA topoisomerase II alpha (*TOP2A*)*,* RAD54 like (*RAD54L*)*,* and X-ray repair cross-complementing 5 (*XRCC5*), as well as the ROS defense-related gene methionine adenosyltransferase 2A (*MAT2A*). When delivered into the HeLa cells, the intracellular level of miR-139-5p became upregulated and promoted apoptosis of cancer cells under radiotherapy. Overall, the synergistic action of all nano-complex components in combination with photodynamic therapy and radiotherapy displayed destruction of neoplasms (Wang et al., 2021b).

Chen et al. (2021) developed a miRNA-conjugated NP treatment for gastric cancer (GC). It is widely known that disturbing mitochondrial-related pathways are an efficient method for inducing apoptosis in gastric tumor cells since GC is considered a mitochondrial metabolic disease. Therefore, to trigger apoptosis via mitochondrion pathways, miR-532-3p was delivered using poly d,l-lactide-*co*-glycolide (PLGA)-PEG NPs labeled with vitamin B12 (VB12), which selectively target the transcobalamin II receptor CD320, overexpressed in GC cells. MiR-532-3p works as a therapeutic oligonucleotide since it inhibits the expression of the apoptosis repressor with the caspase recruitment domain (*ARC*). Consequently, once the ARC restraint effect has been suppressed, the pro-apoptotic protein BAX enters the mitochondria, decreasing mitochondrial membrane potentials, activating ROS production, and promoting mitochondrial permeability transition pores opening (Chen et al., 2021). On the whole, all these events result in mitochondrial damage that promotes the activation of caspase-dependent cell apoptosis. As expected, *in vivo* and *in vitro* tests displayed inhibition of GC cells proliferation after the miRNA treatment, suggesting that miR-532-3p delivered by PLGA-PEG-VB12 NPs regulates the ARC/Bax/mitochondria signaling pathway to cause apoptosis (Chen et al., 2021).

One of the principal disadvantages of radiotherapy relies on the fact that this method becomes less efficient due to radioresistance triggered by DNA damage repair and hypoxia. To avoid a limited response from cells to radiotherapy, radiosensitizers such as miR-181a were delivered into cancerous esophageal squamous cells using 2D graphdiyne-cerium oxide (GDY-CeO_2_) nanozymes. As a result, miR-181a directly bound to the transcripts of its target gene RAD17 checkpoint clamp loader component (*RAD17*), responsible for regulating checkpoint kinase 2 (Chk2) pathway, causing *RAD17* downregulation in radioresistant cells and the overexpression of Chk2 protein (Zhou et al., 2021). Importantly, the downregulation of RAD17 protein prevented DNA repair, therefore enhancing DNA-damage-induced apoptosis, while the accumulation of Chk2 inhibited cancer cell proliferation. Particularly, GDY-CeO_2_ exhibited a better catalase activity by transforming H_2_O_2_ into O_2_ when surrounded by a weakly acidic tumor microenvironment, which caused the mitigation of tumor hypoxia (therefore reversing radioresistance), promoted radiation-induced DNA damage, inhibited tumor growth, and enhanced radiotherapy sensitivity in esophageal squamous cell carcinoma (ESCC) (Zhou et al., 2021).

Ghaffari et al. (2021) reported a PEGylated niosomal formulation capable of simultaneously loading miR-15a and miR-16–1 which was subsequently delivered into the prostate cancer cell line PC3. Niosomes are biodegradable cationic lipids made of synthetic non-ionic surfactants with advantages such as low immunogenicity, cost-effective production, stable structure, and no cytotoxicity. Further, the cationic charges found on their lipid bilayer allow strong electrostatic interactions with miRNAs, resulting in a high loading efficiency. Therefore, the miR-15a and miR-16-1-loaded nioplexes significantly decreased the expression of *BCL2* augmenting the degree of cell death in PC3 cells. As well, nioplexes loaded with both miRNAs showed a higher cellular uptake compared to the NPs loaded with one miRNA alone (Ghaffari et al., 2021).

Yan et al. (2022a) designed a multifunctional chemical/gene-therapeutic-loaded nanomaterial that restrains epithelial ovarian cancer cell (SKOV_3_) development by detecting and reversing drug resistance, along with treating cancer in a chemical-photothermal manner. Concretely, these researchers embedded Cyanine 5 (Cy5)-modified miRNA let-7i and platinum (IV) onto a nano-graphene oxide platform (NGO), which was transported into the cisplatin-resistant ovarian cancer cell line SKOV3DDP*.* Let-7i was employed owing to its well-known capacity to negatively regulate cyclin D1, a protein involved in cell proliferation. Besides, the attached fluorophore Cy5 was employed as a probe to detect mRNAs related to drug resistance when miRNAs were bound to them. On the other hand, platinum (IV) was included in the formulation since it gets converted into the chemotherapeutic particle platinum (II) under the stimuli of glutathione (GSH) in the acidic and reductive tumor microenvironment. Moreover, folate was included in the nanocomposite to achieve targeted delivery to drug-resistant cancer cells. NGO photothermal properties were activated via NIR laser radiation, which significantly enhanced the therapeutic efficiency of this bio-responsive nanocomposite. Ultimately, Cy5-modified miRNA, folate and platinum-loaded PEGylated nano-graphene oxide complex (^Cy5−miR^NGO-PEG-FA-Pt) showed outstanding stability, remarkable drug uptake capacity, and great proficiency to induce cytotoxic effects in cisplatin-resistant carcinoma cells (Yan et al., 2022a).

Soon after, miR-320 was proposed as a promising miRNA to treat head and neck cancer (HNC). This miRNA is naturally downregulated in HNC cells and stimulates apoptosis, represses angiogenesis, counteracts drug resistance, and inhibits cancer progression. However, its delivery into HNC cells has been challenging due to its poor cellular penetration, degradation, and inefficient endosomal escape. Thereby, to overcome such drawbacks, Lo et al. (2022) delivered miR-320 into human tongue squamous carcinoma cell lines (SAS) and SAS-bearing mice using cationic solid lipid NPs (SLNs) coated with a combination of trans-activator (TAT) and peptides containing the asparagine-glycine-arginine motif (NGR). This peptide combination, characterized by cysteine-rich transcriptional activation domains and arginine-rich RNA binding motifs was named T-peptide for efficiency purposes. Accordingly, T-peptide provided SLNs with tumor-homing moiety and mediated cell penetration; this initial miRNA-containing NP activated diverse signaling pathways, leading to the inhibition of drug resistance-related mechanisms. As a result, it sensitized HNC cells for the subsequent NP. Further, with the purpose of enhancing miR-320 therapeutic impact, chemotherapy drug oxaliplatin (Oxa) was delivered through liposomes covered with T-peptide and a nuclear-localization-sequence containing peptide (R-peptide) to provide the nanocarrier with cell- and nucleus-penetrating capability. Oxa is a platinum-related compound that impedes DNA replication, transcription, and repair in cancer cells by triggering a DNA cross-linkage at the N_7_ positions of adenine and guanine (Lo et al., 2022). SLNs and liposomes were sheathed with polyglutamic acid and polyethylene glycol (PGA-PEG) to make the nanocarriers pH-responsive. In this way, the NPs’ cargoes were optimally released when exposed to the acidic tumor environment. Overall, *in vivo* and *in vitro* assays demonstrated satisfactory uptake of both Oxa and miR-320, decreased Oxa-associated toxicities (i.e., neutropenia and thrombocytopenia), and antitumor activity (Lo et al., 2022).

As previously stated, radiotherapy has been associated with low oxygen concentrations in the tumor microenvironment. To overcome this issue in rectal cancer, zeolitic imidazolate framework-8 (ZIF-8) nano-complexes were built to deliver nano-MnO_2_ particles and miR-181a mimics together into mouse colon cancer cell line MC38 and MC38-bearing mice. ZIF-8 was chosen as the nanocarrier because of its controlled component release, excellent biodegradability, and negligible toxicity (Hao et al., 2022). Findings showed that MnO_2_ NPs reacted with endogenous H_2_O_2_ to form O_2_, which led to a reduction in hypoxia and subsequent sensitivity to radiotherapy. Further, MnO_2_ considerably blocked the scavenging activity of glutathione on hydroxyl groups by transforming this compound to glutathione disulfide, therefore magnifying the apoptotic efficacy of chemodynamic therapy. Additionally, induced miR-181a overexpression triggered DNA damage after radiotherapy via double-strand breaks, hence working as a potential inhibitor of angiogenesis and tumor growth. Moreover, the expression of HIF1-*α* was found to be significantly reduced. On the whole, tumors showed increased radiation sensibility, inhibited proliferation, decreased migration, and apoptosis improvement (Hao et al., 2022).

Oncogenic miR-30a-5p has received particular attention for the treatment of ocular melanoma. Concretely, this miRNA downregulates *E2F7*, responsible for promoting the proliferation, migration, and apoptosis of carcinogenic cells. Under such premises, this nucleic acid was released into MUM2B, CRMM2, and CM 2005.1 ocular melanoma cell lines, as well as into MUM2B-bearing nonobese diabetic/severe combined immunodeficiency (NOD/SCID) mice (Ma et al., 2023). To achieve optimal transportation, the chosen delivery vehicles were GSH-responsive quasi-mesoporous magnetic nanospheres (MMNs), since they protect the miRNA from nuclease degradation and promote M1-like macrophages to secrete pro-inflammatory cytokines that induce apoptosis. Furthermore, NP-derived Fe^2+^ reacted with H_2_O_2_ produced by M1 macrophages to generate Fe^3+^, OH-, and free OH radicals, promoting apoptosis. Overall, the outcomes of this inquiry suggest that miR-30a-5p can be efficiently delivered via GSH-responsive MMNs, blocking *E2F7* and *caspase-3* expression, thus facilitating the inhibition of ocular melanoma and induced apoptosis both *in vivo* and *in vitro* (Ma et al., 2023). The main findings regarding the application of nanotechnology-mediated delivery of miRNA-based drugs in these other types of cancer are listed in Table 1.

**TABLE 1:** Summary of the main therapeutic effects achieved with the delivery of miRNA-based therapeutics through nanotechnological devices in different types of cancer.

Cancer	Nanoparticle	Artificial miRNA delivered	Co-delivered drug(s)	Biological model	Main therapeutic effects	Reference
Liver cancer	Multivalent rubber-like RNA NPs	miR-122	PTX	HepG2 cells and hepatocellular carcinoma mice xenograft	Silencing of drug exporters and oncogenic proteins, as well as inhibition of tumor growth	Wang et al. (2021a)
	LA-PAMAM	miR-218	-	HepG2 cells and mice model	Decreased tumor progression and improved liver histological features	Elfiky et al. (2021)
Bladder cancer	Cancer cell membrane coated calcium carbonate NPs	miR-451	Adr	BIU-87/Adr cells and BIU-87/Adr tumor-bearing Balb/c nude mice	Inhibition of multidrug resistance and increased accumulation of intracellular Adr with anticancer effects	Wei et al. (2020)
Ovarian cancer	Omentum-derived exosomes	miR-199a-3p	-	OC cell lines and OC mice model	Inhibition of cell proliferation and invasion	Kobayashi et al. (2020)
Cervical cancer	SiO_2_-polyethyleneimine NPs	miR-let-7c-5p	-	HeLa cells	Inhibition of cell proliferation and migration	Shao et al. (2020)
Colon cancer	CXCR4-targeted polymeric NPs coated with poly-L-glutamic acid	miR-200c	Dab	Murine colon carcinoma cells (MC-38) and MC-38 tumor-bearing mice	Enhanced immune responses against tumors	Nguyen et al. (2021)
Cancer in general	R9 modified with^125^I-labeled RGD and Ce6	miR-139-5p	-	HeLa cells and mouse xenograft tumor models	Enhanced the radiotherapy sensitivity with minimal toxicity	Wang et al. (2021b)
Gastric cancer	PLGA-PEG-VB12 NPs	miR-532-3p	-	BGC-823 cells and BGC-823-tumor-bearing mice	Mitochondrial damage, increased apoptosis, and inhibition of cell proliferation	Chen et al. (2021)
Esophageal cancer	GDY-CeO_2_ nanozymes	miR-181a	-	KYSE30 and KYSE180 cells, and mice model	Alleviation of tumor hypoxia, enhancement of radiation-induced DNA damage, and inhibition of tumor gowth	Zhou et al. (2021)
Prostate cancer	Cationic PEGylated niosomes	miR-15a and miR-16–1	-	PC3 cells	Increased apoptosis of cancer cells	Ghaffari et al. (2021)
Cancer in general	Nano-graphene oxide platform	let-7i	Platinum (IV)	Cisplatin resistant SKOV3 cells	Reversed intracellular drug and enhanced chemical-photothermal therapy	Yan et al. (2022a)
Head and neck cancer	SLNs coated with a combination of TAT and peptides containing the NGR motif	miR-320	Oxa	Human tongue squamous carcinoma SAS cells and SAS-bearing mice	Decreased Oxa-associated toxicities and maximized antitumor efficacy	Lo et al. (2022)
Rectal cancer	ZIF-8 nano-complexes	miR-181a	MnO_2_	MC38 and MC38-bearing mice	Increased radiosensitivity, inhibited proliferation, decreased migration, and enhanced apoptosis	Hao et al. (2022)
Ocular melanoma	MMNs	miR-30a-5p	-	Cancer cells and mice model	Enhanced pro-inflammatory antitumor immunity against melanoma	Ma et al. (2023)

## 4 Concluding remarks

The complexity of the pathophysiology of cancer and its widespread incidence in the world population has greatly accelerated the design of innovative therapeutics in recent years. As a result, nanocarrier-mediated miRNA drug delivery has emerged as a worthwhile alternative for safe and effective cancer treatment. In this regard, several reports have provided clear evidence that NPs offer a number of advantages when delivering artificial miRNAs (alone or along with chemotherapy drugs), such as controlled release, increased stability, low toxicity, and targeted delivery. In addition, the results observed in cancer cell lines and animal models, including the induction of apoptosis, inhibition of tumor growth and development, avoidance of metastasis, as well as increased sensitivity to radiotherapy or chemotherapy, have clearly demonstrated the prospective use of nanotechnology in miRNA-based cancer therapeutics. Nevertheless, more pre-clinical and clinical studies are needed to ensure the effectiveness and safety of the reported nanoformulations.

## 5 Future perspectives

As noted in this review, molecular biologists have been working tirelessly to investigate the prospective applications of nanotechnology-mediated delivery systems for miRNA drugs. Yet, a number of hurdles and unanswered questions remain to be addressed in future research. As a matter of fact, one of the key requirements for these types of nano drugs to reach the pharmaceutical market is that rigorous assessments must be done to determine the physicochemical, pharmacokinetic, and pharmacodynamic properties of these devices, as well as their efficacy and safety (Sun et al., 2020). Toxicological assessments are mandatory to ensure that these therapies do not generate adverse health effects. In this context, developing *in silico* models to evaluate miRNA nanomedicines would be desirable to forecast clinical responses before their extrapolation to *in vitro* or *in vivo* models (Allan et al., 2021; Ansari et al., 2021).

One of the key challenges in cancer treatment is the resistance to chemotherapeutic drugs and radiotherapy that many patients manifest (Bukowski et al., 2020; Olivares-Urbano et al., 2020). Some examples of miRNAs involved in the modulation of chemoresistance are let-7, miR-1, miR-21, miR-34a, miR-122, miR-155, and miR-223 (Mondal et al., 2022). Different studies have elucidated that miRNAs can work as promoters or inhibitors of radioresistance (Podralska et al., 2020; Cabrera-Licona et al., 2021). MiRNAs associated with radioresistance in different types of cancer comprise miR-21, miR-124, miR-181a/d, miR-150, miR-155, miR-196a, miR-221, miR-222, among others; while miR-18a, miR-18a-5p, miR-132, miR-133a, and miR-145 have been related to radiosensitivity (Mohammadi et al., 2021). Accordingly, these miRNAs could be the therapeutic targets of upcoming studies in which they can be administered through NPs to increase the efficacy of chemotherapy and/or radiotherapy, as demonstrated in some of the articles reviewed here.

It is important to highlight that, in recent years, advanced techniques have been employed for the production of NPs from plants, which could be used as biocompatible tools in cancer therapy (Karmous et al., 2020; Yu et al., 2020). Intriguingly, plant-derived NPs offer numerous advantages, including cultivability, economic feasibility, high stability, fast synthesis, good scalability, appropriate size, and low toxicity (Gulia et al., 2022). Moreover, both plant viruses (e.g., cowpea mosaic virus [CPMV], potato virus X [PVX], and tobacco mosaic virus [TMV]) and plant virus-like particles have been harnessed as NPs in multiple studies to deliver therapeutic agents against cancer (Shoeb et al., 2021; Venkataraman et al., 2021). Even so, to the best of our knowledge, plant-made NPs have been barely utilized in miRNA-based drug delivery; hence their application in this field should be further explored. Especially to develop plant-derived edible NPs containing either antagomirs or miRNA mimics, along with other anti-cancer compounds (Iravani and Varma, 2019).

Another aspect that remains to be thoroughly analyzed during the delivery of artificial miRNAs lies in the co-delivery of miRNA-centered treatments in conjunction with phytochemicals that can magnify the restoring effects of the same. In light of this, Kang et al. (2021a) proved that the co-delivery of curcumin and miR-144-3p through heart-targeted extracellular vesicles has a significant effect on improving myocardial infarction. In such a study, miR-144-3p was chosen as the co-delivered miRNA since it has been previously detected as one of the key contributors to the therapeutic effects triggered by curcumin (Kang et al., 2021a). Although these types of synergistic devices have not yet been explored in cancer miRNA nanomedicine, several studies have elucidated the inferences of phytochemicals on the positive or negative regulation of miRNAs associated with the development or inhibition of cancer. For example, in different cancers, phytochemicals such as curcumin, galangin, and silibinin were found to downregulate the expression of miR-21. On the other hand, miR-34, miR-122, miR-133a, and miR-145 have been identified as tumor suppressor miRNAs regulated by curcumin, oleanolic acid, brazilein, and isorhapontigenin, respectively (Son et al., 2020). Consequently, the NP-driven delivery of these therapeutic combinations with summative effects should be investigated henceforth.

**FIGURE 6:**
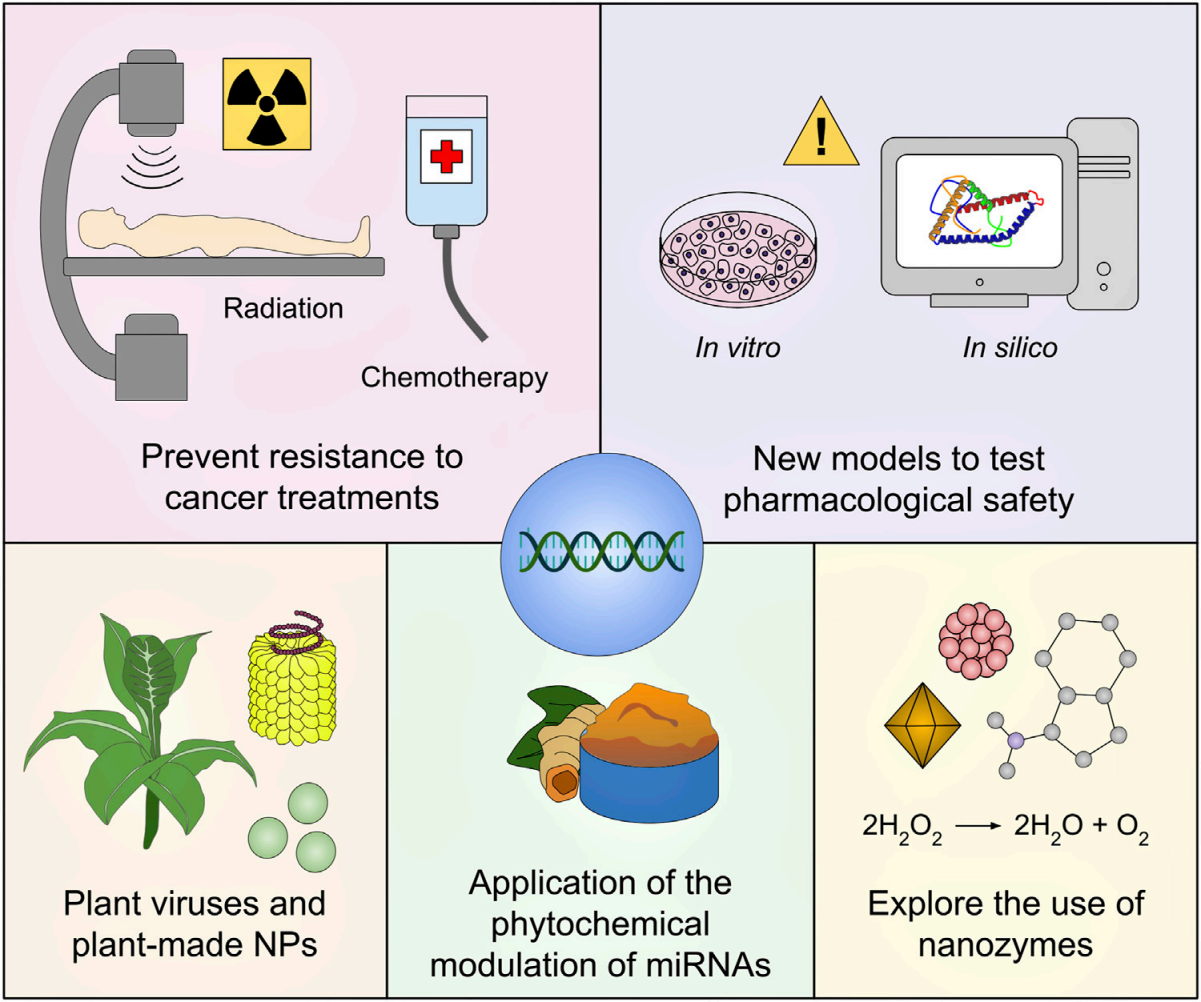
Future insights for nanotechnology-mediated miRNA-based therapeutics in cancer treatment. One of the key challenges in cancer treatment relies on the fact that patients commonly develop resistance to therapies such as chemotherapy and radiation, miRNAs capable of modulating this response should be exploited as therapeutic targets. Furthermore, the development of new *in silico* models to allow early pharmaceutical and biosafety studies of miRNA-centered nanomedicines would be highly desirable. Plant NPs, as biocompatible and cost-effective devices, should be leveraged for the delivery of artificial miRNAs along with plan viruses and plant virus-associated particles. Likewise, forthcoming studies should explore the combination of NPs with phytochemicals that modulate miRNA expression in cancer to generate innovative treatments. As well, nanozymes, being great immunomodulators, could be used in combination with miRNA-centered drugs to improve antitumor effects.

As well, nanozymes, which are catalytic NPs with natural enzyme-mimicking properties, have emerged as prospective materials for cancer immunotherapy. Indeed, nanozymes not only serve as drug delivery systems but also as enzymes that modulate the immunosuppression and oxidative stress of the tumor microenvironment via catalytic activities (e.g., catalase, peroxidase, superoxide dismutase, and oxidase) (Phan et al., 2022; Zhang et al., 2022). Nanozymes displaying catalase activity are useful to produce oxygen (required for O_2_-dependent photodynamic therapy and radiotherapy) from endogenous hydrogen peroxide (H_2_O_2_), whereas peroxidase mimetic nanozymes transform H_2_O_2_ into water and ROS (which also increases the effectiveness of photodynamic therapy) (Thangudu and Su, 2021). Nonetheless, the use of nanozymes together with artificial miRNAs has been scarcely assessed, see Zhou et al. (2021). Therefore, nanozymes should be exploited in upcoming research to compliment the impact of miRNA-based cancer therapies. These future directions are depicted in Figure 6.

Undeniably, the manipulation and delivery of miRNAs via NPs have considerably expanded and revolutionized the therapeutic landscape in the field of oncology, positioning them as potential candidates for novel approaches in cancer treatment. More profound research into this subject will open the gateway to elucidating the underlying molecular mechanisms of cancer initiation and progression linked to the miRNA-mediated gene regulatory networks. To date, pre-clinical studies have established the potential of miRNA-based cancer treatments as viable therapeutic targets in cancer therapy. Furthermore, the utilization of delivery nanoplatforms has demonstrated improved cell specificity and controlled release, resulting in enhanced effectiveness. However, it is crucial to exercise caution and avoid overestimating these therapeutic strategies. Comprehensive validation of their safety, efficacy, pharmacokinetics, pharmacodynamics, biodistribution, and ethical considerations in human subjects is necessary before drawing definitive conclusions and recommending widespread clinical implementation.

As a matter of fact, achieving efficient and selective delivery of such therapeutic agents to tumor cells remains a formidable task. The complexity arises from the heterogeneity of tumors, where different cells within the tumor microenvironment can exhibit distinct characteristics (Yang et al., 2021a). To overcome these hurdles, the development of nanocarriers with enhanced targeting capabilities to specifically reach and penetrate tumor cells while sparing healthy tissues is mandatory. Recent studies have focused on various strategies, including surface modifications of nanocarriers with targeting ligands, utilization of stimuli-responsive systems, and incorporation of tumor-specific antibodies or peptides, aptamers, vitamins, and carbohydrates to improve tumor targeting efficiency (Bilal et al., 2021; Taleghani et al., 2021). Though progress has been made in these areas, comprehensive assessments are still needed to optimize and validate these strategies. The development of novel targeting ligands with high specificity and affinity, as well as the refinement of stimuli-responsive systems, requires further investigation. Additionally, the translation of these findings from pre-clinical models to clinical applications requires rigorous evaluation and validation.

Likewise, another critical aspect of nanocarrier-mediated delivery of miRNA is the biodistribution of the formulation and its potential side effects. After administration, nanocarriers usually distribute throughout the body, interacting with various tissues and organs. Consequently, it is crucial to carefully assess the biodistribution profile to ensure minimal off-target effects and toxicity. Recent advancements in imaging techniques, such as positron emission tomography (PET), magnetic resonance imaging (MRI), and fluorescence imaging, have provided valuable support for tracking the biodistribution of nanocarrier systems in pre-clinical and clinical settings (Murar et al., 2022; Salih et al., 2023). Moreover, enhancing biocompatible and biodegradable nanocarriers to facilitate efficient clearance from the body and reduce the potential long-term accumulation-related adverse effects is a matter that deserves further exploration (Yan et al., 2020; Anwar et al., 2021).

Last but not least, one critical aspect to consider when designing miRNA-based drugs against cancer is the potential for a single miRNA to target multiple mRNAs (Inoue and Inazawa, 2021). Current studies using miRNA nanoformulations mostly focus on well-characterized cancer-associated miRNAs. However, a number of such reports didn´t ensure the absence of undesired effects caused by the pleiotropic activity of the employed miRNA drug. In addition to the above, it should be noted that cell cultures are commonly used to test the miRNA-based drugs via NPs, and it does not offer a complete picture of how these drugs affect gene expression in other cells (although the chances are low due to the specificity of NPs for cancer cells). Therefore, to enhance the efficacy of miRNA-based drugs, researchers must broaden their investigations to ensure the absence of undesired off-target effects related to miRNAs targeting of multiple mRNAs in targeted- and healthy cells.

Undeniably, significant work remains to be done in the field of miRNA nanoformulations for cancer since only two clinical studies are registered on ClinicalTrials.gov (https://clinicaltrials.gov/) in this arena, which are a lipid NP-formulated miR-193a-3p mimic (named INT-1B3) against advanced solid tumors (NCT04675996) and EnGeneIC Delivery Vehicles (EDVs) combined miR-16 mimic (named TargomiRs) for malignant pleural mesothelioma and NSCLC (NCT02369198). These studies, along with other registered clinical trials related to cancer miRNA-centered therapeutics are shown in Table 2.

**TABLE 2:** Clinical trials in the field of miRNA-based therapeutics against cancer.

Title of the study	Type of cancer	Targeted miRNA	Description of the drug	Status	Identifier
First-in-Human Study of INT-1B3 in Patients With Advanced Solid Tumors	Advanced solid tumors	miR-193a-3p	INT-1B3 is a miR-193a-3p mimic delivered via lipid NPs	Recruiting	NCT04675996
MesomiR 1: A Phase I Study of TargomiRs as 2nd or 3rd Line Treatment for Patients With Recurrent MPM and NSCLC	Malignant pleural mesothelioma and NSCLC	miR-16	TagomiRs are miR-16 mimics delivered via EDVs, which are considered non-living bacterial minicells (NPs)	Completed	NCT02369198
A Multicenter Phase I Study of MRX34, MicroRNA miR-RX34 Liposomal Injection	Primary liver cancer, lymphoma, melanoma, multiple myeloma, renal cell carcinoma, NSCLC, and SCLC	miR-34a	MRX34 is a liposomal miR-34a mimic	Terminated	NCT01829971
Pharmacodynamics Study of MRX34, MicroRNA Liposomal Injection in Melanoma Patients With Biopsy Accessible Lesions (MRX34-102)	Advanced melanoma	miR-34a	MRX34 is a liposomal miR-34a mimic	Withdrawn	NCT02862145
SOLAR: Efficacy and Safety of Cobomarsen (MRG-106) vs Active Comparator in Subjects With Mycosis Fungoides (SOLAR)	Cutaneous T-cell lymphoma, mycosis fungoides subtype	miR-155	Cobomarsen is an inhibitor of the activity of miR-155	Terminated	NCT03713320
PRISM: Efficacy and Safety of Cobomarsen (MRG-106) in Subjects With Mycosis Fungoides Who Have Completed the SOLAR Study (PRISM)	Cutaneous T-cell lymphoma, mycosis fungoides subtype	miR-155	Cobomarsen is an inhibitor of the activity of miR-155	Terminated	NCT03837457

In conclusion, it is not difficult to anticipate that miRNA-based cancer therapeutics will be accelerated in the future decade. Relevantly, the innovation of COVID-19 mRNA vaccines over the last 3 years (Curreri et al., 2023) has presented clear evidence that next-generation RNA-centered molecular pharmaceutics could offer the key to substantial disease-modifying therapies. Particularly, the fact that the Food and Drug Administration (FDA) recently approved an RNA lipid NP formulation that contains a siRNA targeting transthyretin (Onpattro) for the treatment of hereditary transthyretin-mediated amyloidosis opens up possibilities for the clinical translation of numerous small RNA-based treatments facilitated via NP delivery (Akinc et al., 2019; Rouge, 2023). Finally, we believe the information presented in this review will help to drive the clinical translation of the nanocarrier-mediated delivery of miRNAs to treat different types of cancer.
