# Supplementary material for: Applications of nanotechnologies for miRNA-based cancer therapeutics: current advances and future perspectives
Source: Front Bioeng Biotechnol. 2023 Jul 27;11:1208547. doi: 10.3389/fbioe.2023.1208547 (PMC10416113; doi:10.3389/fbioe.2023.1208547)
Supplement: Supplementary file 1 [file Table1.DOCX]

**Table 1** Summary of the main therapeutic effects achieved with the delivery of miRNA-based therapeutics through nanotechnological devices in different types of cancer.

| **Cancer** | **Nanoparticle** | **Artificial miRNA delivered** | **Co-delivered drug(s)** | **Biological model** | **Main therapeutic effects** | **Reference** |
| --- | --- | --- | --- | --- | --- | --- |
| Liver cancer | Multivalent rubber-like RNA NPs | miR-122 | PTX | HepG2 cells and hepatocellular carcinoma mice xenograft | Silencing of drug exporters and oncogenic proteins, as well as inhibition of tumor growth | (Wang et al., 2021a) |
|  | LA-PAMAM | miR-218 | - | HepG2 cells and mice model | Decreased tumor progression and improved liver histological features | (Elfiky et al., 2021) |
| Bladder cancer | Cancer cell membrane coated calcium carbonate NPs | miR-451 | Adr | BIU-87/Adr cells and BIU-87/Adr tumor-bearing Balb/c nude mice | Inhibition of multidrug resistance and increased accumulation of intracellular Adr with anticancer effects | (Wei et al., 2020) |
| Ovarian cancer | Omentum-derived exosomes | miR-199a-3p | - | OC cell lines and OC mice model | Inhibition of cell proliferation and invasion | (Kobayashi et al., 2020) |
| Cervical cancer | SiO_2_-polyethyleneimine NPs | miR-let-7c-5p | - | HeLa cells | Inhibition of cell proliferation and migration | (Shao et al., 2020) |
| Colon cancer | CXCR4-targeted polymeric NPs coated with poly-L-glutamic acid | miR-200c | Dab | Murine colon carcinoma cells (MC-38) and MC-38 tumor-bearing mice | Enhanced immune responses against tumors | (Nguyen et al., 2021) |
| Cancer in general | R9 modified with ^125^I-labeled RGD and Ce6 | miR-139-5p | - | HeLa cells and mouse xenograft tumor models | Enhanced the radiotherapy sensitivity with minimal toxicity | (Wang et al., 2021b) |
| Gastric cancer | PLGA-PEG-VB12 NPs | miR-532-3p | - | BGC-823 cells and BGC-823-tumor-bearing mice | Mitochondrial damage, increased apoptosis, and inhibition of cell proliferation | (Chen et al., 2021) |
| Esophageal cancer | GDY-CeO_2_ nanozymes | miR-181a | - | KYSE30 and KYSE180 cells, and mice model | Alleviation of tumor hypoxia, enhancement of radiation-induced DNA damage, and inhibition of tumor gowth | (Zhou et al., 2021) |
| Prostate cancer | Cationic PEGylated niosomes | miR-15a and miR-16-1 | - | PC3 cells | Increased apoptosis of cancer cells | (Ghaffari et al., 2021) |
| Cancer in general | Nano‐graphene oxide platform | let-7i | Platinum(IV) | Cisplatin resistant SKOV3 cells | Reversed intracellular drug and enhanced chemical-photothermal therapy | (Yan et al., 2022a) |
| Head and neck cancer | SLNs coated with a combination of TAT and peptides containing the NGR motif | miR-320 | Oxa | Human tongue squamous carcinoma SAS cells and SAS-bearing mice | Decreased Oxa-associated toxicities and maximized antitumor efficacy | (Lo et al., 2022) |
| Rectal cancer | ZIF-8 nano-complexes | miR-181a | MnO_2_ | MC38 and MC38-bearing mice | Increased radiosensitivity, inhibited proliferation, decreased migration, and enhanced apoptosis | (Hao et al., 2022) |
| Ocular melanoma | MMNs | miR-30a-5p | - | Cancer cells and mice model | Enhanced pro-inflammatory antitumor immunity against melanoma | (Ma et al., 2023) |
