# Supplementary material for: Applications of nanotechnologies for miRNA-based cancer therapeutics: current advances and future perspectives
Source: Front Bioeng Biotechnol. 2023 Jul 27;11:1208547. doi: 10.3389/fbioe.2023.1208547 (PMC10416113; doi:10.3389/fbioe.2023.1208547)
Supplement: Supplementary file 2 [file Table2.DOCX]

**Table 2** Clinical trials in the field of miRNA-based therapeutics against cancer.

| **Title of the study** | **Type of cancer** | **Targeted miRNA** | **Description of the drug** | **Status** | **Identifier** |
| --- | --- | --- | --- | --- | --- |
| First-in-Human Study of INT-1B3 in Patients With Advanced Solid Tumors | Advanced solid tumors | miR-193a-3p | INT-1B3 is a miR-193a-3p mimic delivered via lipid NPs | Recruiting | NCT04675996 |
| MesomiR 1: A Phase I Study of TargomiRs as 2nd or 3rd Line Treatment for Patients With Recurrent MPM and NSCLC | Malignant pleural mesothelioma and NSCLC | miR-16 | TagomiRs are miR-16 mimics delivered via EDVs, which are considered non-living bacterial minicells (NPs) | Completed | NCT02369198 |
| A Multicenter Phase I Study of MRX34, MicroRNA miR-RX34 Liposomal Injection | Primary liver cancer, lymphoma, melanoma, multiple myeloma, renal cell carcinoma, NSCLC, and SCLC | miR-34a | MRX34 is a liposomal miR-34a mimic | Terminated | NCT01829971 |
| Pharmacodynamics Study of MRX34, MicroRNA Liposomal Injection in Melanoma Patients With Biopsy Accessible Lesions (MRX34-102) | Advanced melanoma | miR-34a | MRX34 is a liposomal miR-34a mimic | Withdrawn | NCT02862145 |
| SOLAR: Efficacy and Safety of Cobomarsen (MRG-106) vs. Active Comparator in Subjects With Mycosis Fungoides (SOLAR) | Cutaneous T-cell lymphoma, mycosis fungoides subtype | miR-155 | Cobomarsen is an inhibitor of the activity of miR-155 | Terminated | NCT03713320 |
| PRISM: Efficacy and Safety of Cobomarsen (MRG-106) in Subjects With Mycosis Fungoides Who Have Completed the SOLAR Study (PRISM) | Cutaneous T-cell lymphoma, mycosis fungoides subtype | miR-155 | Cobomarsen is an inhibitor of the activity of miR-155 | Terminated | NCT03837457 |
